# Feasibility of a prospective cohort study to support future investigation of prognostic factors for venous leg ulcer healing

**DOI:** 10.1136/bmjopen-2026-122867

**Published:** 2026-10-12

**Authors:** Sara Lloyd-John, Joe Harvey, Nichola J Barron, Rachel E B Watson, Adam J Reid, Jason K F Wong, Sarah E Herrick, Jo C Dumville

**Affiliations:** 1 Division of Musculoskeletal and Dermatological Sciences, School of Biological Sciences, The University of Manchester Faculty of Biology Medicine and Health, Manchester, UK; 2 Manchester University NHS Foundation Trust, Manchester Academic Health Science Centre, Manchester, UK; 3 Division of Cell Matrix Biology and Regenerative Medicine, School of Biological Sciences, The University of Manchester Faculty of Biology Medicine and Health, Manchester, UK; 4 A*STAR Skin Research Laboratory (A* SRL), Agency for Science, Technology and Research (A*STAR), Skin Research Institute of Singapore, Singapore; 5 Department of Plastic Surgery & Burns, Manchester University NHS Foundation Trust, Manchester, UK; 6 Division of Nursing, Midwifery & Social Work, School of Health Sciences, The University of Manchester, Manchester, UK

**Keywords:** VASCULAR MEDICINE, WOUND MANAGEMENT, Prognosis, Community-Based Participatory Research

## Abstract

**Objectives:**

To evaluate the feasibility of recruitment, longitudinal clinical data collection and repeated dressing-sample collection within routine NHS community venous leg ulcer centres, to inform a future larger prospective cohort study evaluating prognostic factors for venous leg ulcer healing.

**Design:**

Prospective, longitudinal feasibility cohort study with repeated measures.

**Setting:**

Three community leg ulcer clinics in Greater Manchester, UK.

**Participants:**

100 adults with venous leg ulcers.

**Interventions:**

Recruitment commenced from 1 August 2023 to 29 July 2024 with follow-up data collection continuing until 2 October 2024 (62 weeks in total). The study integrated two key components: use of the ComplexWounds@Manchester Biobank as a supporting research infrastructure, and implementation of a novel biomarker methodology involving wound fluid collection from patient dressings. Outcome measures were monitored until the venous leg ulcer healed or until the end of the study.

**Outcome measures:**

Participant recruitment, participation, feasibility of collecting baseline and follow-up clinical data, healing outcomes, biological sampling and participant retention.

**Results:**

Of 101 eligible patients, 99% consented, 100 participants were recruited over a period of 53 weeks. With the exception of body mass index data (missing for 11 participants), there were minimal missing baseline data. During the study, 74 participants achieved complete healing, with 10 participants remaining unhealed at study end, 4 lost to follow-up, 9 withdrawn from clinic and 3 withdrawn from the study. Biological sampling was feasible with a mean of five samples collected per participant. Dressing practices were heterogeneous, with 45 dressing types used in total. The median time taken from the Biobank’s collection of the last dressing sample of the day, transport and processing before storage in the laboratory for all clinic sessions was 85 min.

**Conclusions:**

Biobank-supported recruitment, longitudinal clinical data collection and repeated dressing-sample collection were integrated into routine community venous leg ulcer clinics with no observed impact on usual standard of care. These findings support the feasibility of larger-scale prospective cohort studies evaluating prognostic factors for venous leg ulcer healing, although future studies should include longer follow-up, recruitment closer to ulcer onset and prespecified progression criteria.

**STRENGTHS AND LIMITATIONS OF THIS STUDY**

- The prospective cohort study design was a strength of this feasibility study, as this approach is ideal for the identification and validation of prognostic factors in real-world populations.
- Research infrastructure is limited in community care settings; therefore, this study utilised the ComplexWounds@Manchester Biobank infrastructure to facilitate clinical data and biological sample collection enabling long-term repeated sampling and allowing expansion for larger studies in the field.
- Monitoring recurrence rates and recruiting participants closer to ulcer inception to capture the full healing process was a limitation and would be desirable to include in future studies.
- While the feasibility of undertaking research using the Biobank facilitated approach was used, further exploration of the feasibility using the same approach for prognostic studies with appropriate sample sizes (informed by this study) is now required.
- Generalisability may be limited by underrepresentation of participants from ethnic minority groups, those whose first language was not English and single-area recruitment.

## Introduction

 Complex wounds are superficial, partial or full-thickness skin defects that heal by secondary intention.^1^ Venous leg ulcers are the most common type of complex wounds in the UK, primarily resulting from underlying chronic venous insufficiency.^2 3^ They are a significant clinical problem and account for 60%–80% of leg ulcers.^2 3^ They occur on the lower limb, heal slowly and frequently recur, with healing and recurrence further affected by age and treatment adherence.^4^ Data across NHS settings in Leeds reported a venous leg ulcer point prevalence of 0.29 (95% CI 0.25 to 0.33) people per 1000,^5^ with similar figures found in Greater Manchester NHS community care services.^6^ Venous leg ulcers significantly impact an individuals’ health-related quality of life, causing pain, increasing infection risk and limiting mobility and social activities.^7 8^ In the UK, venous leg ulcer management mainly occurs in the community, often involving frequent appointments with community nurses, which incurs substantial costs for healthcare services.^6^

A key outcome for people with venous leg ulcers is complete wound healing; people would like their wounds to be dressing-free and to know how long this will take.^9–11^ Time taken for these wounds to heal is also important information for healthcare systems, service planning and care optimisation. As venous leg ulcer healing can be highly variable, prediction of individual healing times requires robust prognostic data,^12–15^ which also considers the use of effective treatments such as compression therapy.^16^ Prognostic factors are key prediction measures and can include clinical factors as well as biological markers (biomarkers) measured from patient samples. Identification of prognostic factors for venous leg ulcer outcomes would improve understanding of likely wound trajectory and facilitate better personalisation of treatment and management plans.

Protein biomarkers in particular, have received attention for their potential as independent prognostic factors for wound healing estimation and as directly modifiable treatment targets to improve venous leg ulcer healing.^12^ Such biomarkers can be measured using various techniques often with wound fluid as the analytical sample.^17–19^ Wound fluid is a heterogeneous complex mixture of plasma proteins, proteases, inflammatory cytokines, growth factors and other products secreted by cells at the wound site which represents the wound microenvironment^20^ and is therefore an ideal sample type for prognostic biomarker measurement being both simple and non-invasive to collect.^21 22^

While there has been an increased interest in the identification of prognostic factors for wounds,^12 13^current data are limited by extremely small sample sizes and poor study design and analyses, particularly when utilising prognostic biomarker measurements.^23^ In a scoping review assessing studies reporting biomarkers in wound fluid, findings were based on small cohorts: of the 270 studies reporting sample size, 139 (52%) included only 1–20 participants.^24^ To investigate prognostic factors, rigorous prospective cohort studies are required; however, these can be costly and complex to facilitate, especially if biological sampling alongside clinical data collection is required.^24^ These issues are especially true for venous leg ulcer prognostic research because community nursing services, where most care is received, have insufficient research infrastructure (such as electronic databases, sample storage, transport facilities and research personnel), in addition to staff capacity and workload.

To address these longstanding issues and promote better prognosis research in wound care, an explorative prospective cohort study design was implemented to assess the feasibility of delivering a larger future study that could lead to impactful benefit for patients and services. The purpose of this study was to test the feasibility of conducting a robust venous leg ulcer cohort study that could operate as part of routine NHS care.^25^ Our study design incorporates exploration of two key elements to support the feasibility of the proposed cohort. First, use of the ‘ComplexWounds@Manchester Biobank’ as a research enabler. Due to the high workloads and limited staffing capacity in many community services and the lack of local research infrastructure, a link is required to bridge the translational gap from clinic to laboratory. Biobanks are ideal in this scenario to provide staff capacity for clinical data and sample collection alongside transport and storage of both electronic records and physical samples. Second, inclusion of a novel method for the collection of wound fluid extracted from patient clinical dressings, which is compatible with various analytical assay methods.^26^ The combined sampling and assay method analysis allows identification and levels of specific proteins in complex wound fluid. This approach aimed to avoid having to undertake time-consuming wound sampling activities such as wound aspiration^27^ or wound swabbing techniques^28^ which are restrictive due to the amount of protein collected and time-consuming if added to standard patient care.

Here, we report the feasibility of embedding recruitment, longitudinal clinical data collection and repeated dressing-sample collection from the same patient within routine NHS community venous leg ulcer clinics. The feasibility data are intended to inform the design and delivery of a larger future prospective cohort study that could evaluate prognostic factors for venous leg ulcer healing. General feasibility questions were explored as well as more specific questions about the use of our sampling approach in practice and the Biobank as an infrastructure for NHS community focused wound care research.

## Methods

### Study design

The study was designed as a prospective, longitudinal feasibility cohort study with repeated measures to assess whether procedures needed for future prognostic-factor studies could be delivered within routine NHS community venous leg ulcer clinics. The study aimed to explore the feasibility of patient recruitment, clinical data access and dressing sample collection with variable follow-up (minimum follow-up time of 3 months or until reference ulcer healed) within NHS clinics in Manchester, while minimising disruption, assessing variation in clinical practices and exploring the ability to record outcomes in routine practice. Recruitment commenced from 1 August 2023 to 29 July 2024 (53 weeks) with follow-up data collections continuing until 2 October 2024 (62 weeks in total). The study was registered and published on the Open Science Framework on 12 January 2024 (Reference: osf.io/3jxmt) (online supplemental material 1).

### Setting and participants

The Lead Nurse in Community Services was contacted initially to discuss the study and to acquire their support in accessing the community leg ulcer clinics. Discussions with the lead nurses of the clinics alongside clinic visits then took place to organise logistics and understand how the clinics were run. Once this was established, patients receiving wound care for venous leg ulcers were recruited from three Manchester University NHS Foundation Trust community leg ulcer clinics. Recruitment, data and sample collection were performed by experienced Biobank research staff and sample transport, storage, processing and analyses were performed by University of Manchester research staff. All data were collected in the Research Electronic Data Capture (REDCap, Software V.15.5.8) tool electronically at the Biobank and samples and clinical data anonymised prior to transfer by Biobank staff to the University of Manchester research staff.

Potential participants were first identified by NHS clinic nurses who provided study details, so patients could decide whether to be involved and meet with the Biobank nurse. Identified patients were confirmed for eligibility based on the inclusion and exclusion criteria and underwent a full consent process before participation in the study.

Participants were deemed eligible if they met all the following criteria:

- Received care via Manchester University NHS Foundation Trust in participating leg ulcer clinics.
- Had at least one venous leg ulcer as defined as any break in the skin which had either (1) been present for >6 weeks or (2) occurred in a patient with a history of venous leg ulceration.^16^ Ulcers were considered purely venous if, clinically, no other aetiology was suspected, were venous in appearance (ie, moist, shallow of an irregular shape) and located wholly or partially within the gaiter region of the leg. Ankle brachial pressure index (ABPI) was required to be >0.8 and for those in whom ABPI was not possible, Toe brachial pressure index (TBPI) >60 mm Hg was used as an alternative to confirm absence of arterial disease. If unable to obtain both ABPI/TBPI, referral to a specialist was made.
- Could understand the requirements of the study and could provide written and verbal informed consent.
- Patients ≥18 years of age.

Participants were excluded if they met the following criteria:

- Unwilling or unable to give informed consent.
- Previous or current enrolment in any study to evaluate a wound-focused treatment option 30 days prior to entry into the study (the details of the other study were discussed and a decision made if they could be recruited).
- People who are currently pregnant.
- Non-English-speaking people.
- Patients <18 years of age.

Participants entering the study were assigned a unique identifier and data were pseudo-anonymised at the point of data collection. Prescreening data were collected at each clinic visit which included the number of patients attending the clinic, the number of ineligible patients and the number of participants recruited to the study.

A formal sample size calculation for this study was not undertaken, but the aim was to recruit 100 participants in the 53-week period, as this was deemed a suitable number to assess feasibility with the available resources.

### Outcome measures

Our primary feasibility outcomes were based on exploration of participant recruitment, study participation, feasibility of collecting baseline and follow-up clinical data, biological sample collection, participant retention and operational feasibility. Specific progression thresholds were not set, as the aim was to explore and describe whether the infrastructure developed and implemented was able to facilitate a cohort study and if so where potential barriers were for iteration. The following feasibility questions were used to guide and structure this exploration:

Recruitment and study participation

- Will patients consent to participate in this type of study?
- How many eligible participants can be recruited in a 53-week period from participating community clinics in Greater Manchester?
- What language translation requirements would be needed for future studies?
- How long does it take to recruit a participant and collect the required baseline data?

Feasibility of collecting baseline and follow-up data

- Can comorbidity data be collected?
- Can the use of compression therapy be recorded in recruited participants to assess how commonly it is used and variation of use?
- Can wound infection events be collected to assess the incidence of wound infection with basic microbiological data to inform future studies?
- Can healing outcomes be recorded and assessed adequately for future studies?

Feasibility of biological sampling

- How often can dressing samples be collected from participants?
- What type of dressings are commonly used in the community for venous leg ulcers and do dressing types vary over time?
- Can collection, storage and transfer of dressing samples for wound fluid protein extraction be carried out in a timely and secure way?
- What is the cost of resources per sample or per participant for collection, storage and processing?

Study retention

- How many people recruited to the study withdrew or were lost to follow-up?

Operational feasibility

- Was Biobank-supported research feasible within routine clinics where NHS care was being delivered?

Secondary feasibility outcomes were to report descriptive clinical findings to assess measurement of feasibility. These included healing proportion, infection frequency, dressing types and compression use.

### Data and sample collection

#### Recruitment and baseline data

Biobank staff attended clinics and identified potential participants via discussions with the NHS clinic nurses. Eligible and ineligible patients were identified and reasons recorded for ineligibility. For those excluded because they were unable to speak English, their primary spoken language was logged. Ulcer presence was an essential eligibility criterion for the study, but because patients may continue attending clinic after healing for compression therapy and follow-up care, many screened patients did not have an active ulcer at assessment. For those who consented and were recruited, baseline demographic data were collected (online supplemental table S1). The reference ulcer, the largest ulcer by area at baseline, was denoted on a leg ulcer diagram to ensure consistent sampling. Baseline wound related information collected for the reference ulcer was location, ulcer duration, ulcer size (to calculate area) and infection status. The types of dressings used (including the dressing name, brand and the number of dressing changes per week) were also captured. All information was collected by Biobank research staff via a combination of asking the participant, consulting the patient’s Leg Ulcer Integrated Care Pathway documentation (MFT 00162, 2022)^29^ completed by the clinic nurses or by the use of electronic patient records (Hive EPR and EMIS, Manchester NHS Foundation Trust) which were accessed either in clinic or off site at the Biobank offices. Collection of data from electronic records reduced data collection times for each participant and minimised disruption to clinical care.^26^

#### Follow-up data collection

Most participants returned to the clinics weekly for standard dressing changes. Biobank research staff consulted participants’ notes or electronic records to determine the number of dressing changes over a weekly period. Sampling times were minimal, with dressings collected directly after removal from the reference ulcer, which took place as part of ulcer management. When the dressing was removed by the clinic nurses, an experienced Biobank staff member collected the dressing in clinic, prepared it for transport and placed it on ice for transfer to the Blond McIndoe and Kellgren Laboratories (Stopford Building, University of Manchester, UK). Once received, the dressing samples were processed, wound fluid extracted and analysed at the laboratory by a University of Manchester researcher. The feasibility assessment for this study was limited to the practical aspects of collecting, transporting, storing and processing dressing samples, rather than the analytical validity of biomarker measurement. Detailed assessment of biomarker quality, assay performance, protein recovery and comparability across dressing types is outside the scope of this paper and will be addressed in future related biomarker-focused work. Wound area measurements (measured by simple ruler method for length and width by clinic nurses), compression status and any changes to participant health data were also recorded at each clinic visit by the Biobank staff (online supplemental table S1). Compression therapy was presented as a binary variable indicating whether compression was being received; however, the type of compression used was also documented. Any missed clinic visits or occurrences of missed data collection were also recorded. At weekly clinical visits the wound area, assessment of healing (NHS clinic nurses confirmed if wound had healed), and incidence of infection (assessed visually by NHS clinic nurses as per the Leg Ulcer Integrated Care Pathway and confirmed by microbiology culture) were logged on REDCap and extracted by the Biobank staff member.

Clinical data and sample collection continued until participant exited the study or conclusion of the study. Participants exited the study due to one of the following reasons: complete reference ulcer healing; withdrawal by participant on request, death, loss to follow-up, withdrawn from clinic, withdrawal from study or study completion. Lost to follow-up meant that the participant did not return to clinic when they missed four clinic visits (8 weeks). Withdrawal from the clinic was a clinical decision unrelated to the study protocol and was due to changes in ulcer management requirements such as discharge from clinical care, transferral to treatment rooms as compression therapy no longer required or receiving at home treatment. Withdrawal from the study was due to change in ulcer aetiology. The date of study exit, and reason for exit, were recorded on REDCap. Complete ulcer healing was defined as ‘complete epithelial cover in the absence of a scab (eschar) with no dressing required’^30^ and was confirmed visually by NHS clinic nurses. The date the NHS clinic nurse considered the reference leg ulcer to have healed was recorded in the patient’s notes and extracted by Biobank research staff. Data were stored in password-protected files on a secure shared drive that was accessible only to authorised study personnel. The participants were assigned a unique identifier, and data were pseudo-anonymised at the point of data collection to be used for data transfer (online supplemental material 2), communication and analysis purposes.

### Data analysis

Descriptive statistics were used to summarise recruitment, data collection, dressing collection and sampling and study retention outcomes using minimum and maximum values, mean and SDs, median and IQRs and counts and percentages.

Recruitment data were presented as those patients meeting the eligibility criteria who did and did not convert to recruitment and those who were ineligible. The cumulative time taken for screening, recruitment and baseline data collection processes to be completed was recorded for a subset of 20 participants from one clinic which was deemed to be sufficient to be representative of the sample population demographically. Descriptive statistics are presented for the quantification of other measures for all participants where available.

When assessing the feasibility of collecting, transferring and storing dressings for wound fluid protein extraction, the average time was calculated as time taken from collection of the last dressing sample, transport and processing before storage in −80°C freezers at the laboratory. Data were presented as mean and SDs, median and IQRs and percentage of missing data. Study costs included Biobank charges, staff time, transport, storage and laboratory consumables, with total consumables and Biobank costs calculated by dividing by the number of samples collected and participants recruited, respectively, to estimate average per-sample and per-participant costs.

Participant retention is defined as the proportion of participants who withdrew or were lost to follow-up and was displayed as counts and percentages. Data analysis was carried out in R Studio V.4.3.1 (R Studio, Boston, Massachusetts, USA).

### Patient and public involvement

A lived experience advisory group comprising individuals with direct experience of venous leg ulcers was established alongside the cohort feasibility study. The group participated in facilitated discussions and contributed to the development of the study through providing recommendations on the wording of participant-facing materials and sharing perspectives on the importance and relevance of the research. The advisory group informed the direction of the project by helping to prioritise and refine the research objectives.

## Results

### Recruitment and study participation

Recruitment commenced from 1 August 2023 to 29 July 2024 with follow-up data collections continuing until 2 October 2024 (62 weeks in total). During the study period, across all clinics, there were 101 eligible patients, 99% of which 100 consented and were recruited (clinic 1: 25%, clinic 2: 10%, clinic 3: 65%) (figure 1). A median of eight participants were recruited per month (95% CI 4 to 12). Clinic 3 had the highest recruitment rate as this was the largest in terms of overall patient numbers (figure 2). The primary reason for exclusion from the study was ineligibility, reasons for ineligibility included the ulcer not being considered venous, lack of capacity to consent, the person being non-English speaking and the absence of an open wound, which was the most common reason for ineligibility (94%) (table 1). One patient with translation needs was identified (spoken language is deliberately not reported here to ensure they are not identifiable, but it was recorded). For a sample of 20 participants, screening, recruitment and baseline data collection took a median time per participant of 24.5 min (95% CI 22 to 26) (online supplemental table S2). Clinic 1 and clinic 2, total patient appointment time periods were 30 min, while clinic 3 allocated 45-minute periods per patient. The time needed for recruitment did not disrupt NHS clinic times and was absorbed into waiting time and the clinic session itself. Follow-up data collection times were not recorded but took considerably less time than initial screening, recruitment and baseline data collection times as less data were required. Disruption was not assessed using a formal measure and this finding was based on researcher observation and informal feedback from clinic staff.

**Figure 1:**
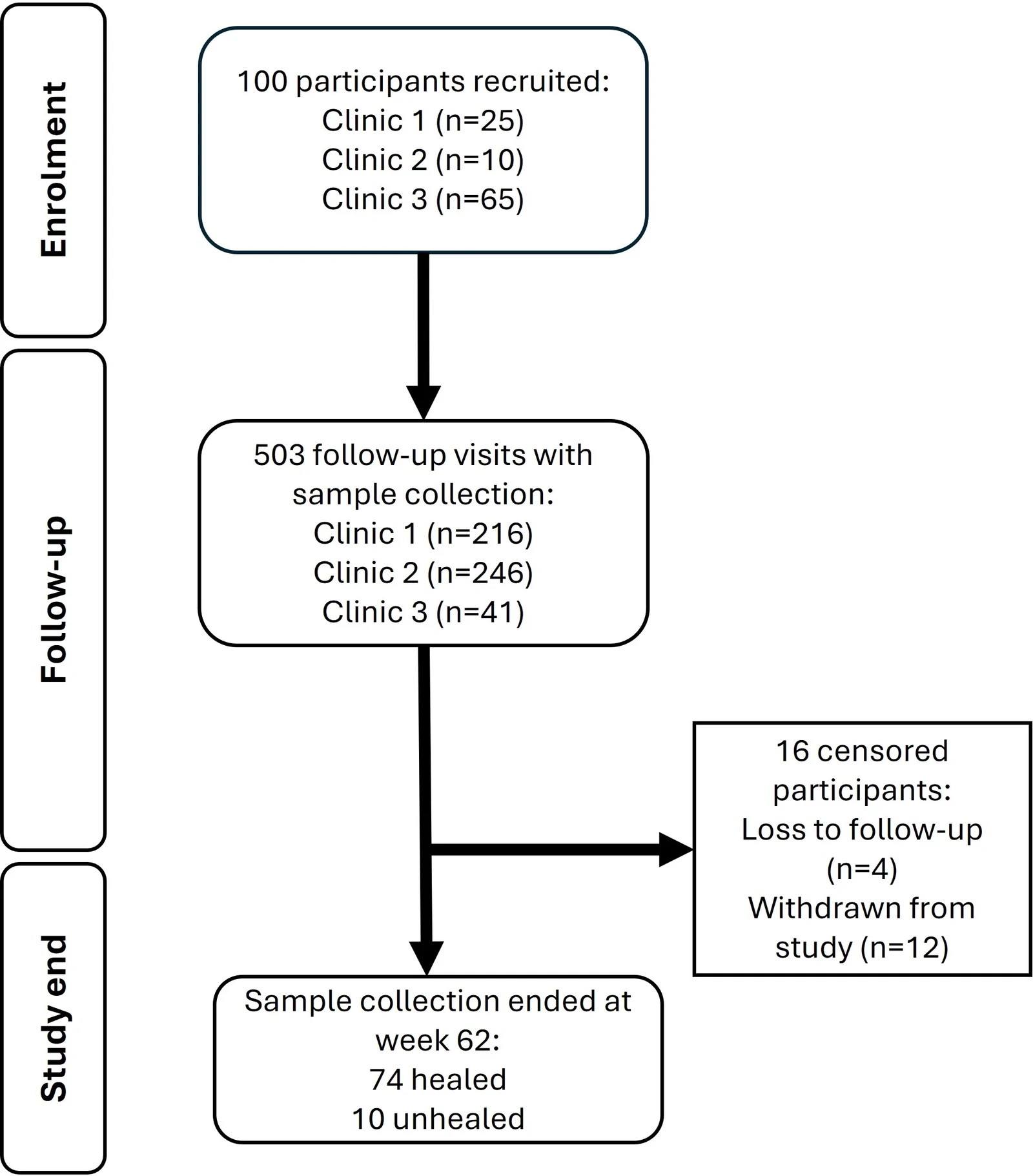
Recruitment flow diagram. Flow diagram showing recruitment, follow-up and outcomes for both clinical data collection and sample collection. During the study period, across all clinics, there were 101 eligible patients, of which 100 consented and were recruited (clinic 1: 25%, clinic 2: 10%, clinic 3: 65%) (figure 1). Clinic 3 had the highest recruitment rate as this was the largest in terms of overall patient numbers.

**Figure 2:**
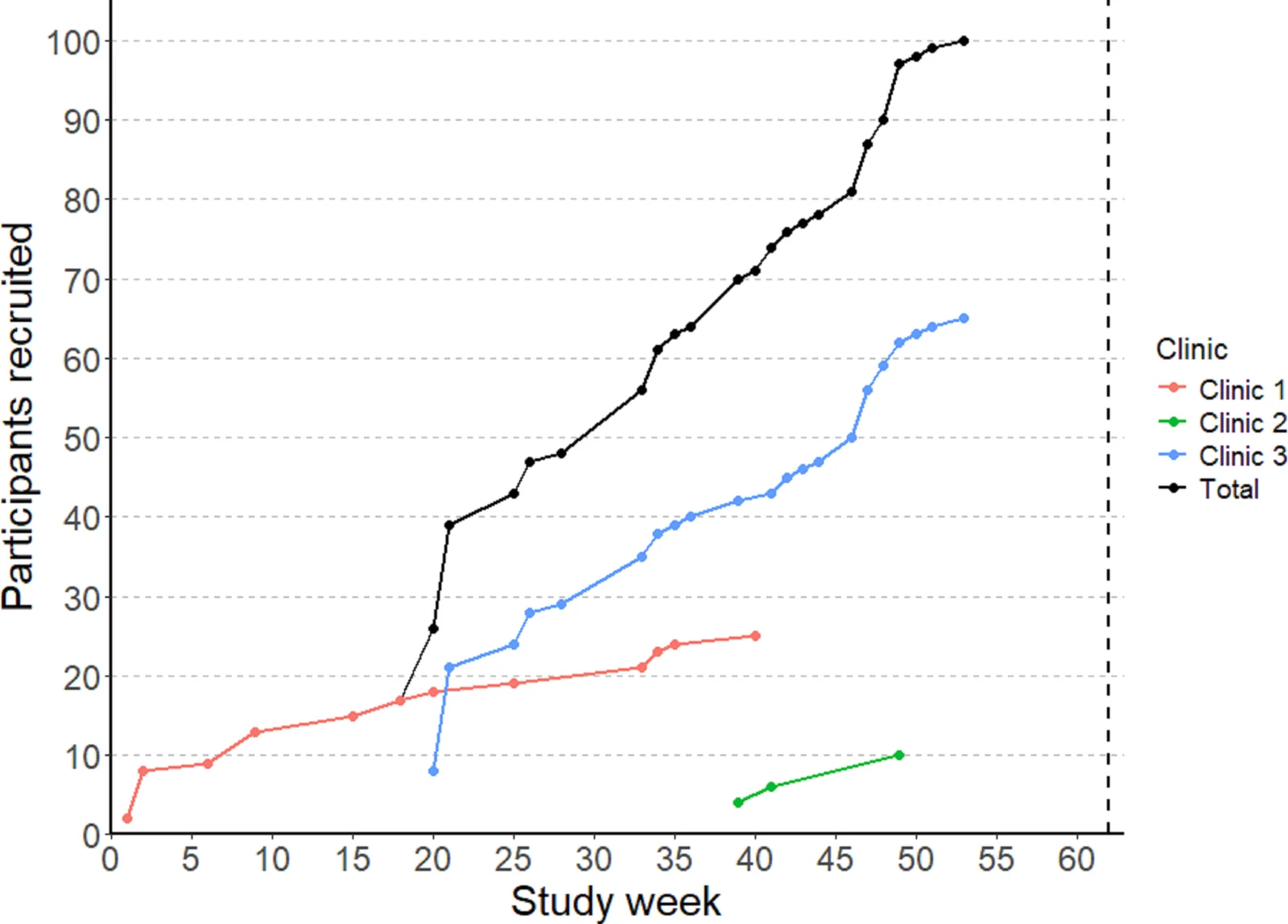
Participants recruited per study week from three community leg ulcer clinics. 100 participants were recruited over the course of 53 weeks and followed up to 62 weeks. The graph displays the number of participants recruited per week from each of the three community leg ulcer clinics and gives a median recruitment rate per month of eight participants (95% CI 4 to 12). The majority of participants were recruited from clinic 3 (65%) as this was the largest leg ulcer clinic compared with clinic 1 (25%) and clinic 2 (10%).

**Table 1:** Eligibility assessment over a 53-week recruitment period*

Assessed for eligibility (%)	1580 (100)
Included	525 (33)
Excluded	1055 (67)
Reasons for exclusion (%)	1055 (100)
Ineligible	896 (85)
Declined to participate	5 (1)
Lack of staff capacity	76 (7)
Other	78 (7)
Ineligibility (%)	896 (100)
No ulcer	845 (94)
Lack capacity to consent	6 (<1)
Non-English speaking	4 (<1)
Other	41 (5)

* Due to data protection laws, individuals are not identifiable pre-enrolment. Therefore, numbers represent each patient visit and so will include multiple counts from some individuals.

### Baseline and follow-up data collection

#### Baseline data

Participants’ demographic data are included in table 2. Participants had a mean age of 69 years (SD: 13 years) with slightly more male than female participants (58% male, 41% female and 1% with missing data). Most participants self-reported being White British (93%). Participants’ mean body mass index (BMI) was 29.2, which is in the overweight range (25–29.9). The most common comorbidities recorded in this cohort of venous leg ulcer patients were hypertension (42%) and cardiovascular disease (34%). Overall, there was little missing baseline data for most fields collected, the exception was BMI where 11 participants (11%) had missing data. Most participants were receiving compression therapy at recruitment (94%), and this was maintained over the study period (97%) with only three participants not receiving compression therapy at all during the study period. The median number of ulcers present at recruitment was one, with 68 participants (68%) having one ulcer, while the maximum number of ulcers a participant presented with was 9. There was considerable variation in wound duration (mean: 6.1 months, SD 8.3 months) which may be due to recruitment occurring at different phases of the wound healing process. Repeated sampling was also limited for several participants, possibly due to being recruited during the latter stages of healing. Wound area (mean: 20.1 cm^2^, SD 38.8 cm^2^) at baseline also showed large variation as several much larger wounds skewed the average. Wound area was measured using a simple ruler method^31^ and although a common and cost-effective method,^32^ it is often subjective and relatively imprecise for wounds with irregular edges, often resulting in overestimation of the wound area compared with other available measurement methods.^33^

**Table 2:** Participant demographic, health and venous leg ulcer information at baseline

Age (years)	n=100
Mean (SD)	69 (13)
Median (IQR)	69 (17.5)
Missing data	0
Sex	n=100
Male (%)	58 (58)
Female (%)	41 (41)
Missing data (%)	1
Ethnicity	n=100
White British (%)	93 (93)
White European (%)	4 (4)
Other (%)	3 (3)
BMI*	n=100
Mean (SD)	29.2 (7.5)
Median (IQR)	28.2 (8.9)
Missing data (%)	11 (11)
Selected comorbidities†‡	n=100
Hypertension (%)	42 (42)
Cardiovascular disease (%)	34 (34)
Diabetes (%)	14 (14)
Cancer (%)	6 (6)
Missing data (%)	1 (1)
Smoking status	n=100
Never smoked (%)	55 (55)
Ex-smoker (%)	29 (29)
Current smoker (%)	15 (15)
Missing data (%)	1 (1)
Mobility	n=100
Mobile (%)	65 (65)
Walking aid (%)	31 (31)
Wheelchair (%)	2 (2)
Missing data (%)	2 (2)
Compression therapy	n=100
Baseline	
Receiving compression therapy (%)	94 (94)
Not receiving compression therapy (%)	6 (6)
Missing data (%)	0 (0)
Over study duration	
Receiving compression therapy (%)	97 (97)
Not receiving compression therapy (%)§	3 (3)
Missing data (%)	0 (0)
Number of ulcers	n=100
Mean (SD)	1.6 (1.3)
Median (IQR)	1 (1.0)
Missing data (%)	0
Duration of reference ulcer (months)¶	n=100
Mean (SD)	6.1 (8.3)
Median (IQR)	3.7 (5.2)
Missing data (%)	2 (2.0)
Reference ulcer area (cm²)	n=100
Mean (SD)	20.1 (38.8)
Median (IQR)	6.1 (17.3)
Missing data (%)	1 (1.0)

* BMI=weight/(height)².

† Some participants may have multiple comorbidities.

‡ Only comorbidities associated with venous leg ulcers occurrence and healing were displayed.

§ Calculated as number of participants who did not receive compression after baseline until study exit.

¶ The reference ulcer was chosen as the largest ulcer by area at baseline wound measurement.

BMI, body mass index.

#### Follow-up data

##### Clinical data

Participants had a total of 503 follow-up visits with most visits at clinic 1 (n=216, 42.9%) and clinic 3 (n=246, 48.9%) (table 3). The mean number of follow-up visits per participant across all clinics was 5 (SD: 5.67, median: 3, IQR: 4). The median length of time participants remained in the study across all clinics was 8 weeks, as seen in clinic 1 (8 weeks, IQR: 16) and clinic 3 (8 weeks, IQR: 8) whereas clinic 2 had a longer median time of 12.5 weeks (IQR: 10.5).

**Table 3:** Follow-up visits and length of time in study in three venous leg ulcer community clinics

Follow-up visits	n=503
Clinic 1 (%)	216 (42.9)
Clinic 2 (%)	41 (8.2)
Clinic 3 (%)	246 (48.9)
Length of time in study*	
All clinics	n=100
Mean (SD) (weeks)	10.9 (9.4)
Median (IQR) (weeks)	8 (9.3)
Clinic 1	n=25
Mean (SD) (weeks)	13 (13.4)
Median (IQR) (weeks)	8 (16.0)
Clinic 2	n=10
Mean (SD) (weeks)	10.7 (7.9)
Median (IQR) (weeks)	12.5 (10.5)
Clinic 3	n=65
Mean (SD) (weeks)	10.1 (7.6)
Median (IQR) (weeks)	8 (8.0)

* Length of time in study calculated from the number of weeks since consenting at baseline to study exit.

### Wound infection

In total, six participants (6%) entered the study with an infection at baseline. Of those who had acquired an infection, the most common infective species was *Pseudomonas aeruginosa* (n=4, 66.6%) (online supplemental table S3). During the study, a further six participants (6%) developed an infection with half of those acquiring *P. aeruginosa* (n=3, 50%).

### Healing of the reference ulcer

Healing outcome data for 100 participants were collected with no missing data. Of the 100 participants recruited, 74% reached complete ulcer healing over the study period.

### Biological sampling

The mean number of dressing samples collected per participant over the study period varied, with a mean average of five samples collected per participant (online supplemental table S4). The number of dressings collected each week differed due to the number of participants in the study at one time and if there were any missed clinic sessions (online supplemental figure S1). The dressing materials and types used varied within and between participants (figure 3), with 45 different types of dressings used over the course of the study. No dressing samples were delayed in processing. The only samples that yielded lower wound fluid extraction volumes were from wounds that were close to healing, as expected.

**Figure 3:**
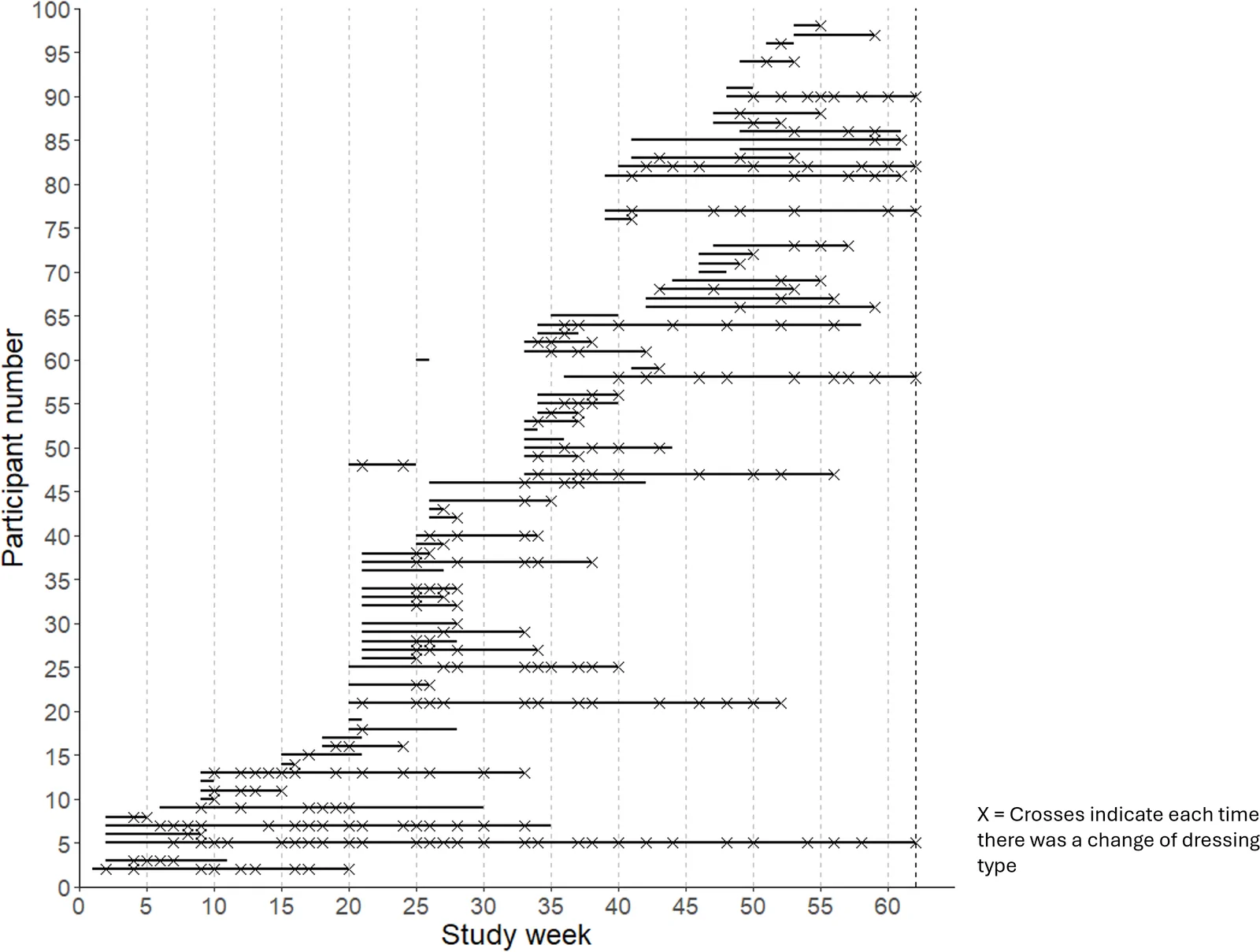
Dressings changes over time for participants from baseline to complete ulcer healing or study exit. Data points were recorded for each dressing collection from all participants (n=100) who entered the study. Dressing collection continued until ulcers healed (n=74), participants were withdrawn from the study (n=3) due to change in ulcer aetiology, withdrawn from clinic (n=9) due to changes in ulcer management requirements such as discharge from clinical care, transferral to treatment rooms as compression therapy no longer required or receiving at home treatment, lost to follow-up (n=4) (the participant did not return to clinic when they missed four clinic visits (8 weeks) or the study ended with the ulcer unhealed (n=10). The study continued for 62 weeks at which point all dressing collections ceased. Dressing types varied within and between participants from week to week due to various factors, for example, exudate level, presence of infection or the size of the wound. There were a total of 758 dressings, and 45 different dressing types were used during the course of the study. Crosses indicate each time there was a change of dressing type.

The median time taken from dressing collection to storage of samples for all clinic sessions was 85 min (95% CI 79 to 91); this did not vary greatly between most sessions or clinics (online supplemental table S5). The study-specific cost per sample resource use is outlined in table 4. The cost per sample was calculated as £65.53 and included collection, transportation, processing and storage only; laboratory biomarker assays were not included in this estimate. The study-specific cost to recruit a participant and collect clinical data at baseline and multiple time points during study involvement via the Biobank was calculated as £157.10 per participant. The total study cost to recruit 100 participants (£15 710.00) and process 503 samples (£32 959.99) was £48 669.99.

**Table 4:** Cost of resources including recruitment, data collection and sample acquisition required for this prospective, longitudinal feasibility cohort study for venous leg ulcers

Resources	Amount per item	Item cost (£)	Total cost for 503 samples (£)
University staff costs			
Laboratory researcher for processing and extraction of dressing samples	2 hours 30 min per sample	Total £53.00 per sample (£21.20 per hour)	£26 659.00
Total			**£26 659.00**
Transport costs			
Mileage (at 45 p per mile) from University of Manchester to:			
Clinic 1–15 miles return Clinic 2–7 miles return Clinic 3–11 miles return	216 visits 41 visits 246 visits	£6.75 £3.15 £4.95	£1458.00 £153.75 £1217.70
Total			**£2829.45**
Laboratory consumables			
Sample bags	503 bags	£2.71 per sample bag	£1363.13
Centrifugal filter units (1 filter per sample)	503 filters	£3.70 each	£1861.10
Buffer 10 mmol Tris (10 mL per sample)	5030mls	£22.40	£22.40
Eppendorf tubes (4×1.5 mL tubes per sample)	2012 tubes	£0.018 per tube	£36.22
Eppendorf tubes (3×0.5 mL tubes per sample)	1509 tubes	£0.025 per tube	£37.73
Freezer sample storage boxes	34 boxes	£4.44 each	£150.96
Total:			**£3471.54**
Storage costs			
Storage of samples in −80° freezer at University of Manchester laboratory	Long term	No cost	No cost
Total			**£0.00**
Total cost for 503 samples			**£32 959.99**
Cost per sample			**£65.53**

Resources	Amount per item	Item cost (£)	Total cost for 1 participant (£)	Total cost for 100 participants (£)
Biobank costs				
Biobank recruitment and sample collection	100 participants	£155.50	£155.50	£15 550.00
Biobank admin management fee (set-up, oversight, invoicing)	1	£160.00	£1.60	£160.00
Total cost per participant			**£157.10**	**£15 710.00**

### Study retention

Of the 100 participants recruited, 74% reached complete ulcer healing (table 5). The remaining 26 participants (26%) were exited from the study either because they remained unhealed at the end of the follow-up period (n=10: 38.5%), lost to follow-up (n=4: 15.4%), were withdrawn from clinic (n=9: 34.6%) due to changes in ulcer management requirements such as discharge from clinical care, transferral to treatment rooms as compression therapy no longer required or receiving at home treatment, or were withdrawn from the study due to change in ulcer aetiology (n=3: 11.5%). Lost to follow-up meant that the participant did not return to clinic when they missed four clinic visits (8 weeks). There were no participants who specifically requested to leave the study after inception.

**Table 5:** Attrition and retention data for 100 venous leg ulcer participants

Endpoint	n=100
Complete ulcer healing (%)	74 (74.0)
Cessation of sample collection (%)	26 (26.0)
Missing data (%)	0
Cessation of study participation	n=26
Unhealed at study end (%)	10 (38.5)
Lost to follow-up (%)	4 (15.4)
Withdrawn from study (%)	3 (11.5)
Withdrawn from clinic (%)	9 (34.6)

## Discussion

### Summary of main findings

Infrastructure has been successfully set up in three NHS community leg ulcer clinics to produce an efficient workflow that could support a future prospective cohort study of prognostic factors in wound fluid derived from dressings. We demonstrated feasibility from recruitment through to dressing collection and transportation for wound fluid sample extraction. The current study demonstrated that the clinical and biological sampling procedures required for such research could be embedded within routine community care. The study appeared acceptable to patients with 100 participants out of 101 eligible patients recruited over a 1-year period consenting to data and sample collection. High retention rates were also achieved, supporting the acceptability of repeated follow-up and sampling procedures in this setting. The workflow was delivered alongside NHS community clinic activity without reported issues or disruptions. Recruitment, clinical data collection and dressing-sample collection were performed using the planned Biobank-supported procedures, indicating that the study processes could be implemented in line with the intended protocol.

The majority of studies carrying out prognostic research alongside biomarker analysis are randomised controlled trials.^34^ Randomised controlled trials achieve faster healing rates when compared with observational cohort studies as they tend to have more restrictive inclusion criteria and therefore, may not be generalisable to the broader venous leg ulcer population.^34^ Cohort studies are therefore better suited for venous leg ulcer prognosis research as they allow for the identification and validation of prognostic factors in real-world populations. Furthermore, they are instrumental in identifying factors associated with healing outcomes. Most leg ulcer management is carried out in community settings, with the majority of the research occurring in secondary care.^5 9^ There are limited studies that have been performed in the community setting which have also involved biological sampling. By comparison with the current study, these have employed small cohort sizes (less than 100 participants), relatively short follow-up periods of less than 6 months^23 26 35–37^ and have shown variability in the outcomes presented.^38–40^ Research infrastructure is generally more limited in community care settings which can cause issues with recruitment and sampling and makes it difficult to standardise sample collection.

Prognostic research in a community setting is essential but has challenges. Models of care differ greatly in the UK where some community settings have specialist wound clinics and others rely on general community nursing. There are differences in frequency of visits, continuity of care, availability of equipment, resource constraints and staffing issues and these variables are difficult to standardise. However, it is vital to conduct research in this setting to address these barriers. The current feasibility study demonstrates that procedures needed to support future prognostic research can be carried out in the community where most venous leg ulcer patients are treated. It is acknowledged that using the infrastructure of the Biobank was instrumental in ensuring that this study was feasible in this setting. The successful collection of biological samples alongside clinical data was achieved and paves the way for future exploration of wound biomarkers with implications for wound trajectory monitoring and analysis of therapeutics.

### Implications and recommendations for future research

These findings can inform the design of a future prospective cohort study to evaluate prognostic factors for venous leg ulcer healing, including decisions about recruitment strategy, follow-up duration, sample collection frequency and resource requirements. Results from this feasibility study can also be used for sample size estimates in larger cohort studies and to other groups by providing an estimate of the variance for the outcome measures.^32^ There is scope to expand further by using the Biobank approach to facilitate different methods of sample collection including swabs while continuing to use the same procedures for recruitment and clinical data collection. In addition, samples from a range of other wound types may be collected, such as arterial/mixed ulcers or diabetic foot ulcers. The Biobank infrastructure therefore provides a flexible and valuable model for exploring a range of wound biomarkers while maintaining consistent clinical data collection.

Additional considerations should be addressed when expanding to sites outside of Greater Manchester. One potential issue is that Biobank facilities may not be available in all areas outside of Greater Manchester. However, there is scope for this Biobank expansion with additional funding, early planning and additional ethical requirements. The Biobank model could be replicated in other geographical locations. The feasibility demonstrated in this study should be interpreted within the context of the specialised Biobank-supported system, rather than feasibility across community services more broadly. Further evaluation in centres with different staffing arrangements, transport distances, clinic workflows and research capacity will be needed before the model can be considered scalable across wider community leg ulcer services. This study demonstrated a median time from dressing collection to sample storage of 85 min. This may vary across different clinical sites and should therefore be accounted for in future studies conducted at multiple locations. This is relevant both because prolonged collection and storage procedures may affect staff workload, and because further evidence is needed to determine whether delays before storage influence the quality or reliability of protein sampling outcome measures.

The main resources identified in this feasibility study were sample transport costs between the clinics and the University of Manchester, laboratory consumables and staffing costs, specifically the Biobank research staff who attended clinics and performed all data and sample collections and the University of Manchester researcher who processed and analysed all dressing samples. An additional cost required for studies involving analysis of biological samples includes laboratory assays, use of extra consumables and staff time. Although such costs are project dependent they may substantially increase the total costs incurred depending on the methods used. Ultimately total cost will vary from study to study with changes to factors such as study duration, frequency of sampling and assay use, these can be modulated to fit within the scope and funding allocations of a particular project.

### Strengths and limitations

This study was strengthened by working with a lived experience advisory group with direct experience of having venous leg ulcers. A further strength was that the study set-up and procedures appeared to be feasible within routine NHS clinic workflows. NHS Trusts outside of Greater Manchester may have different clinic times and structures; thus, it would be essential to take this into account. Future studies should consider incorporating a more structured assessment of intervention burden and disruption to routine clinical practice.

High recruitment and retention rates were an additional strength of this study, nevertheless, recruitment was limited to patients who could attend clinics and not those receiving home care. Patients unable to attend clinics are usually less mobile and may be unwell, and so their addition could potentially impact the population sample in this study. In the future, it would also be useful to monitor recurrence rates and aim to recruit participants closer to ulcer inception to capture the full healing process, which would likely be more feasible in clinics with higher patient capacity and with an expanded recruitment period. Inclusion criteria could then be restricted by wound duration to ensure an adequate sample collection period was achieved.

Another strength of this study was that the age range, male/female ratio and ethnicities of the enrolled participants matched closely with epidemiological data collected from NHS community and primary care services for venous leg ulcer patients in the UK.^5 6^ However, a limitation was that there may be underrepresentation of ethnic minority groups which should be explored in a future larger study. The English-language eligibility criterion was pragmatic for this feasibility study but may have limited participation among people who speak languages other than English, with implications for inclusivity and generalisability across those populations. The high consent rate in this study should also be interpreted in this context, as eligible participants may have faced fewer communication barriers than would be expected in more linguistically diverse settings. In this study, participants were recruited from three community leg ulcer clinics within a single NHS Trust and therefore may not fully represent patients managed in other geographical areas, service models or healthcare systems. In addition, these clinics were associated to established Biobank infrastructure; consequently, the feasibility observed in this study may not be directly transferable to centres without comparable research infrastructure, staffing or wound care pathways.

A limitation in this study also relates to the use of the ruler method to measure wound area, which may have introduced an element of measurement error. Future studies could incorporate digital wound imaging to record wound area and characteristics. This approach would also support independent endpoint verification.^41^ However, the ruler approach reflected standard practice in the community clinics and helped maintain consistency with routine clinical assessment. Similarly, wound healing was assessed by the leg ulcer clinic nurse responsible for each participant’s routine visit. These nurses were experienced in leg ulcer assessment and management, so this approach was consistent with the study’s aim of evaluating feasibility in a real-world community clinic setting, rather than introducing additional adjudication or formal standardisation procedures beyond usual care. In this study, feasibility was not assessed against prespecified progression criteria for recruitment, retention, data completeness or sampling; therefore, conclusions regarding feasibility are partly interpretative and dependent on the aims, scale and design of any subsequent study. However, this reflects the early-stage and exploratory nature of the work, which aimed to generate empirical evidence to inform the design and planning of future prognostic research in venous leg ulcer healing, rather than to make a binary decision about progression to a predefined definitive study.

## Conclusions

Infrastructure has successfully been set up in three NHS community leg ulcer clinics to produce an efficient workflow from recruitment through to dressing collection, transportation and sample extraction. Recruitment and retention rates were high and there were no disruptions to the normal clinic procedures and clinic times. Issues with dressing sample collection and transportation were minimal and scale-up in recruitment and sample collection is feasible using the current methods. The recruitment and collection workflow set up should facilitate long-term repeated sampling and allow expansion of recruitment centres for larger studies in this field. Overall, this feasibility study suggests that a future prospective cohort study evaluating prognostic factors for venous leg ulcer healing could be delivered within routine community care, although further investment and refinement would be required. Future large-scale studies should include longer follow-up, recruitment closer to ulcer onset and prespecified progression criteria.

## Supplementary material

10.1136/bmjopen-2026-122867 online supplemental file 1

10.1136/bmjopen-2026-122867 online supplemental file 2

10.1136/bmjopen-2026-122867 online supplemental file 3

10.1136/bmjopen-2026-122867 online supplemental file 4

## Data Availability

Data are available on reasonable request.
