# Supplementary material for: Feasibility of a prospective cohort study to support future investigation of prognostic factors for venous leg ulcer healing
Source: BMJ Open. 2026 Oct 12;16(10):e122867. doi: 10.1136/bmjopen-2026-122867 (PMC13662055; doi:10.1136/bmjopen-2026-122867)
Supplement: Supplementary file 1 [file bmjopen-2026-122867.reviewer_comments.pdf]

PEER REVIEW HISTORY

BMJ Open publishes all reviews undertaken for accepted manuscripts. Reviewers are asked to complete a checklist review form and are provided with free text boxes to elaborate on their assessment. These free text comments are reproduced below.

ARTICLE DETAILS

Title (Provisional)

Feasibility of a prospective cohort study to support future investigation of prognostic factors for venous leg ulcer healing: findings from a prospective, longitudinal feasibility cohort study with repeated measures

Authors

Lloyd-John, Sara; Harvey, Joe; Barron, Nichola J; Watson, Rachel E; Reid , Adam J; Wong, Jason; Herrick, Sarah E; Dumville, Jo

---

VERSION 1 - REVIEW

**Reviewer**	**1**
**Name**	**Aimanan, Karthigesu**
**Affiliation**	**Universiti Malaysia Sabah**
**Date**	**25-Jun-2026**
**COI**	**None**

This manuscript addresses an important area of wound care research by evaluating the feasibility of conducting a prospective cohort study to identify prognostic factors for venous leg ulcer healing in a community setting. The concept of integrating a biobank infrastructure with routine clinical care is novel and is one of the main strengths of the study.

My main concern is that the manuscript does not clearly define what constituted a successful feasibility study before recruitment began. Although a number of feasibility questions are listed, there are no predefined progression criteria or thresholds for recruitment, retention, sample collection, or data completeness. As a result, the conclusion that the study was feasible is based largely on the authors' interpretation rather than objective criteria. This would be strengthened by referencing established guidance for feasibility studies and stating what outcomes would have been considered acceptable.

The manuscript would also benefit from a clearer separation between feasibility outcomes and descriptive clinical findings. Results such as healing rates, infection rates, and median

healing time are interesting, but they are secondary observations and should not be interpreted as evidence of feasibility. The emphasis should remain on whether recruitment, follow-up, data collection, and repeated sampling were achievable within routine community practice.

There are also some issues with the statistical reporting. The Kaplan-Meier curve estimates the probability of healing over time rather than the hazard rate, and this terminology should be corrected throughout the manuscript. It would also be helpful to provide confidence intervals for the main descriptive outcomes, particularly recruitment, healing, and infection rates.

The generalisability of the findings should be discussed in more detail. Recruitment was limited to three clinics within a single NHS Trust, and patients receiving home-based care and non-English speakers were excluded. These factors may introduce selection bias and limit the applicability of the findings to other healthcare settings. Similarly, the very high consent rate is impressive but may not be reproducible in a larger multicentre study.

The reported median healing time should also be interpreted with caution, as participants entered the study at different stages of ulcer duration. Measuring healing from the time of recruitment rather than ulcer onset is likely to underestimate the true healing time for many patients, and this limitation deserves greater emphasis in the discussion.

Although the study is presented as a prognostic biomarker study, no biomarker data are reported. In reality, this manuscript evaluates the feasibility of collecting biological samples rather than investigating biomarkers themselves. The title and abstract should reflect this more clearly to avoid misleading readers.

Finally, the cost analysis is useful but would benefit from additional detail. It would be helpful to describe how staff costs, transport, laboratory consumables, and overheads were calculated, as this information would assist other groups planning similar studies.

Overall, I think this is a well-conducted feasibility study with clear practical value. The work demonstrates that prospective cohort research with repeated wound fluid sampling can be embedded successfully within routine community venous leg ulcer services. With clearer reporting of feasibility criteria, more accurate statistical terminology, and a fuller discussion of the study's limitations and generalisability, I believe the manuscript would be suitable for publication in BMJ Open.

**Reviewer**	**2**
**Name**	**Thapar, Ankur**
**Affiliation**	**Anglia Ruskin University - Chelmsford Campus, School of Medicine and Medical Technology Research Centre**
**Date**	**17-Jul-2026**
**COI**	**None**

Thank you for allowing me to review this manuscript.

Please use the term feasibility cohort study throughout rather than interchanging it with pilot study - no associations have been drawn.

For those in whom ABPI is not possible how was absence of arterial disease confirmed?

Please state the primary feasibility q first in the aims.

There were a lot of people ineligibile because they did not have an ulcer? Was this not an essential criteria?

PPI group could expand to include a caregiver or spouse.

It is creditable that the patients were recruited bearing in mind the extra time per NHS nurse. Could NIHR primary care nurses help in future trials?

Wound measurement using a ruler is rather basic. Suggest use ImitoMeasure (WoundGenius) to record and measure area and characteristics in future studies. This also allows independent endpoint verification.

Is one clinician at one timepoint enough to adjudicate wound healing?

How was the pseudomonas treated?

**Reviewer**	**3**
**Name**	**Mashili, Fredirick**
**Affiliation**	**Muhimbili University of Health and Allied Sciences, Physiology**
**Date**	**22-Jul-2026**
**COI**	**None**

The authors aimed to assess the feasibility of conducting a prospective cohort study embedded within routine NHS community care to support future investigation of prognostic factors for venous leg ulcer healing. They conducted a single-group, repeated-measures feasibility study involving 100 adults recruited from three community leg ulcer clinics in

Greater Manchester. The study examined recruitment, participation, clinical data collection, repeated dressing-sample collection, sample transportation and processing, participant retention, and the ability to document healing and other clinical outcomes. The authors concluded that prospective cohort research incorporating repeated biological sampling could be embedded successfully within routine community venous leg ulcer care and that the model could support larger prognostic studies.

Overall, this is a potentially valuable manuscript. Research involving venous leg ulcers is commonly conducted in community settings where infrastructure, staffing capacity, sample storage, transport systems and research support may be limited. Demonstrating that longitudinal clinical data and repeated biological samples can be collected alongside routine care is therefore relevant. The high recruitment rate, repeated dressing-sample collection, use of Biobank infrastructure, reporting of operational costs, and apparent lack of disruption to routine clinic procedures are important strengths.

However, several conceptual and reporting issues need to be addressed before the manuscript can be considered scientifically clear and internally consistent. My main concern relates to the mismatch between the title, stated purpose, outcome measures, results and conclusions. The manuscript is presented in several places as though it investigated prognostic factors for venous leg ulcer healing. However, the study was primarily designed to assess the feasibility of conducting a future prognostic cohort study. No prognostic-factor associations, prognostic models, or validation analyses are presented. The manuscript should therefore be revised so that its identity as a feasibility study is maintained consistently throughout. The title, objective, outcome measures, abstract results, conclusion, article summary and discussion should clearly distinguish between:

• The feasibility outcomes assessed in the current study;

• The descriptive clinical outcomes collected to test whether they could be measured; and

• The prognostic analyses intended for a future definitive study.

Major Comments:

1. Mismatch between the title and the actual study purpose

The title is:

Feasibility of a prospective cohort study to investigate prognostic factors for venous leg ulcer healing.

This wording may give the impression that prognostic factors were investigated in the current study. However, the Methods section states that the study was designed to assess the feasibility of running a larger cohort study that would subsequently explore prognostication.

The current study therefore assessed the feasibility of conducting future prognostic research and not investigating prognostic factors directly. No associations between candidate prognostic factors and healing outcomes are analysed, and no prognostic model is developed or validated.

The authors should revise the title to reflect the actual study purpose more accurately.The title should avoid implying that prognostic factors were evaluated unless such analyses are included.

2. The abstract objective combines the current feasibility aim with the future prognostic aim

The abstract objective states:

“To assess the feasibility of conducting a robust prospective cohort study embedded within routine NHS community care to identify prognostic factors for venous leg ulcer healing.”

This sentence combines the immediate purpose of the current study with the intended purpose of a future study. Consequently, it is unclear whether the current study aimed to assess feasibility, identify prognostic factors, or both.

The objective should distinguish these two levels explicitly. For example:

To assess the feasibility of conducting a prospective cohort study, embedded within routine NHS community care, that could subsequently evaluate prognostic factors for venous leg ulcer healing.

Alternatively:

To evaluate the feasibility of recruitment, longitudinal clinical data collection and repeated biological sampling for a future prospective study of prognostic factors for venous leg ulcer healing.

This distinction should also be maintained consistently in the introduction, methods, discussion and conclusion.

3. Feasibility outcomes are not clearly separated from descriptive clinical outcomes

The abstract lists the following outcome measures:

“Participant recruitment, participation, clinical activity, time to healing estimation, dressing sample collection and participant retention.”

Some of these are clearly feasibility outcomes, including recruitment, participation, sample collection and retention. However, “clinical activity” is vague, and “time to healing estimation” is primarily a clinical outcome unless it is explicitly framed as an assessment of whether healing data could be collected adequately for a future study.

The Methods section provides a more extensive list of feasibility questions, but these are not organised into clearly defined primary and secondary feasibility outcomes. The authors should distinguish between:

• Recruitment and participation feasibility: eligibility, consent, recruitment rate, recruitment time and translation requirements.

• Operational feasibility: integration into clinic workflow, disruption to routine care, staff requirements, data access and Biobank support.

• Data-collection feasibility: completeness of baseline and follow-up data, ability to record compression use, infection, wound area and healing.

• Biological-sampling feasibility: number and frequency of dressing samples, transport time, processing, storage and cost.

• Retention and follow-up feasibility: attrition, reasons for exit, duration of follow-up and completeness of healing ascertainment.

• Descriptive clinical outcomes: time to healing, healing proportion, infection frequency, dressing types and compression use.

Clinical findings can be reported, but they should be clearly described as exploratory or descriptive outcomes collected to assess measurement feasibility rather than as substantive prognostic findings.

4. The study lacks clearly prespecified feasibility criteria or progression thresholds

The manuscript lists several feasibility questions, but it does not appear to provide prespecified criteria for determining whether the study would be considered feasible. For example, no thresholds are presented for:

• An acceptable recruitment rate;

• An acceptable consent rate;

• Minimum retention;

• Acceptable levels of missing data;

• Minimum number of dressing samples;

• Acceptable transport and processing time;

• Acceptable cost;

• Acceptable disruption to routine clinic procedures; or

• The proportion of participants with complete healing ascertainment.

Without prespecified progression criteria, the conclusion that the study was feasible is based largely on post hoc interpretation.

The authors should clarify whether formal feasibility criteria were specified in the protocol or registration. If they were, these should be reported and the findings evaluated against them. If they were not, this should be acknowledged as a limitation. The authors should avoid presenting feasibility as an entirely objective conclusion when the decision criteria were not defined in advance.

5. The abstract results overemphasise descriptive clinical findings

The Results section of the abstract gives substantial prominence to median time to healing, the proportion healed, baseline wound infection, the organisms identified and the number of dressing types used.

Although these findings are informative, the abstract should prioritise the outcomes that directly answer the feasibility objective. These include:

• The number and proportion of eligible participants recruited;

• The time required for screening, consent and baseline assessment;

• Participants retention and reasons for study exit;

• The completeness of clinical data;

• The number of repeated dressing samples successfully collected;

• Transport and processing time;

• Operational costs;

• The ability to integrate the procedures into routine clinics; and

• Whether routine care was disrupted.

The finding that Pseudomonas aeruginosa was the most commonly identified organism among a very small number of infected participants is not central to the principal feasibility question. Given the limited abstract word count, it could be removed or moved to the main Results section.

Similarly, the finding that 45 different dressing types were used is relevant mainly because dressing heterogeneity may affect the future use of wound-fluid biomarkers. This relationship should be made explicit rather than presenting dressing heterogeneity as a standalone clinical result.

6. "Minimal attrition" and "missing data were negligible" should be replaced with numerical reporting

The abstract states that retention was high, attrition was minimal and missing data were negligible. These terms are subjective and are not supported by prespecified thresholds.

The authors should report the relevant numbers directly. For example:

- 4 participants were lost to follow-up;
- 3 were withdrawn from the study;
- 9 were withdrawn from the clinic;
- 10 remained unhealed at the end of follow-up;
- No participants specifically requested withdrawal after recruitment;
- Healing outcome data were complete;
- BMI was missing for 11% of participants; and
- Missing for most other variables was low.

This would provide a more transparent account than the terms "minimal" or "negligible." It is also important to distinguish active participant withdrawal from other forms of study exit.

7. Retention is not clearly defined and may be conflated with healing-related study exit

The manuscript reports high retention, but 74% of participants exited after healing, while the remaining participants exited for several different reasons. In a wound-healing study, successful healing is an expected endpoint rather than evidence of retention in the conventional sense.

The authors should define retention carefully. Retention could refer to the proportion of participants who remained under observation until one of the following:

- Confirmed healing;
- Planned end of follow-up; or
- A valid documented clinical exit.

Participants lost to follow-up should be distinguished from participants who exited because of healing, transfer of care, discharge from clinic or study completion.

The reported median participation duration of eight weeks should also not be interpreted automatically as evidence of high retention because follow-up duration was partly determined by healing time and recruitment stage. A participant recruited near the end of the healing trajectory may have a short study duration despite being fully retained.

8. Time to healing should be interpreted cautiously because recruitment did not begin at ulcer onset

The manuscript reports a median time to healing of 10 weeks from recruitment. However, participants were recruited at different stages of ulcer development and healing, with substantial variation in wound duration at baseline. Therefore, this estimate is not the total time from ulcer onset to healing.

This limitation is acknowledged in parts of the manuscript, but it should be made more explicit wherever the healing estimate is presented, especially in the abstract. The preferred wording would be:

Median time from study recruitment to documented healing was 10 weeks.

The authors should avoid wording that could be interpreted as the natural healing duration of venous leg ulcers. Recruitment closer to ulcer onset and restriction or stratification by ulcer duration may be needed in a future prognostic study.

9. The conclusion should focus on what the study directly demonstrated

The abstract conclusion begins:

“Identifying prognostic factors could improve individualised treatment and management…”

This is background justification rather than a conclusion derived from the study. The current study did not identify prognostic factors or demonstrate improved personalised management.

The conclusion should begin with the feasibility findings. For example:

Biobank-supported recruitment, longitudinal clinical data collection and repeated dressing-sample collection could be embedded within routine community venous leg ulcer clinics without apparent disruption to usual care. The study provides operational information for a larger prospective prognostic cohort, although longer follow-up, recruitment closer to ulcer onset and prespecified progression criteria will be required.

The final claim that the study establishes a model for identifying prognostic biomarkers should also be moderated. The study demonstrates the feasibility of the collection infrastructure, but not that the collected samples will necessarily be analytically valid across heterogeneous dressings or clinically useful for prognosis.

10. The article summary does not adequately reflect the feasibility design

The first strength states that the observational prospective cohort design is ideal for identifying and validating prognostic factors. This describes the rationale for the future definitive study rather than a strength of the current feasibility study.

The strengths and limitations should instead focus on what was tested in the present study. Possible strengths include:

- Recruitment through routine community clinics;
- High consent among eligible participants;
- Repeated clinical and biological sample collection;
- Use of established Biobank infrastructure;
- Recording of operational time and resource costs;
- minimal apparent disruption to routine care; and
- complete ascertainment of healing status.

Important limitations include:

- Absence of prespecified feasibility thresholds;
- Recruitment at different stages of wound healing;
- Variable and sometimes short follow-up;
- Exclusion of non-English-speaking patients;
- Exclusion of patients receiving home-based care;
- Recruitment from only three clinics in one geographical area;
- Potential dependence on specialised Biobank infrastructure;
- Underrepresentation of ethnic minority groups;
- Use of routine ruler-based wound measurement;
- Dressing heterogeneity; and
- Limited assessment of recurrence.

The article summary should be revised to distinguish current study achievements from future prognostic ambitions.

11. The role of wound-fluid biomarker analysis requires clearer explanation

The manuscript states that dressing samples were processed and wound fluid extracted and analysed for a separate biomarker study. However, the present manuscript does not report biomarker quality, concentration, assay performance or comparability across dressing types.

The authors should clarify the scope of the present paper. Did the feasibility assessment include only successful collection, transportation and storage, or did it also include evidence that the extracted wound fluid was analytically usable?

This is important because successful physical collection of a dressing sample does not necessarily demonstrate the feasibility of valid biomarker measurement. Different dressing materials may absorb, bind, dilute or alter proteins differently. Given that 45 dressing types were used, the authors should explain whether dressing type could affect protein recovery and whether this will require standardisation, adjustment or validation in the future definitive study.

If analytical validity is outside the scope of this paper, the authors should state this clearly and avoid implying that the complete biomarker pathway has been demonstrated to be feasible.

12. The generalisability of the Biobank-supported model should be discussed more critically

The manuscript concludes that this model could support expansion to additional centres. However, the feasibility observed here depended heavily on experienced Biobank research staff who attended clinics, collected data and samples, arranged transportation, stored records and coordinated laboratory processing.

This infrastructure may not be available in many community settings. The authors acknowledge this issue, but it should be treated as a central limitation rather than only a consideration for future scale-up.

The manuscript should distinguish between:

• Feasibility within a specialised Biobank-supported system; and

• Feasibility within routine community clinics without equivalent infrastructure.

The current findings primarily establish the former. Replication in settings with different staffing, transport distances, clinic structures and research capacity will be necessary before broader generalisability can be claimed.

13. Exclusion of non-English-speaking participants needs stronger consideration

Non-English-speaking patients were excluded, and one potentially eligible patient with translation needs was identified. Although the number was small in this sample, the exclusion criterion has implications for inclusiveness and generalisability.

A future definitive study should consider translated participant materials and interpreter support. The authors should discuss whether language-related exclusion could become more important when recruitment expands to more ethnically and linguistically diverse areas.

The study’s very high consent rate should also be interpreted in the context of this eligibility restriction.

14. The methods should distinguish study design from feasibility purpose

The manuscript alternates between describing the study as:

• A prospective longitudinal cohort study;

• An observational prospective cohort study; and

• A single-group repeated-measures feasibility study.

These descriptions are not necessarily incompatible, but the hierarchy should be clear. The study appears to be a prospective longitudinal feasibility cohort study with repeated measurements.

The authors should use one consistent description throughout the abstract, methods, article summary and discussion. Calling it simply an observational cohort study may overemphasise its prognostic design and understate its feasibility purpose.

15. The discussion should avoid implying that prognostic research was successfully conducted

Several passages state that the study demonstrates that prognostic research can be carried out in the community. More precisely, the study demonstrates that procedures required for a future prognostic study—recruitment, longitudinal data collection and repeated sample collection—can be implemented in these clinics.

The authors should revise statements that imply that valid prognostic-factor research has already been completed. The present study did not evaluate candidate predictors, estimate their associations with healing, assess model performance or validate a prognostic model.

The distinction is important because feasibility of data and sample collection is necessary but not sufficient to establish feasibility of the full prognostic research programme.

Minor Comments:

1. The title should be revised so that it does not imply that prognostic factors were investigated in the current study.

2. In the abstract objective, separate the immediate feasibility aim from the future prognostic aim.

3. Replace "clinical activity" in the abstract outcome measures with a more specific description.

4. Consider using the term "descriptive clinical outcomes" for healing, infection, wound characteristics, compression use and dressing type.

5. Report "time from recruitment to healing" rather than "time to healing" throughout.

6. Replace subjective terms such as “high retention,” “minimal attrition” and “negligible missing data” with numerical results or prespecified criteria.

7. Clarify whether the reported 99% consent rate refers only to eligible patients who were approached.

8. Explain why the absence of an exudating wound was treated as a reason for ineligibility when this criterion is not clearly included in the listed eligibility criteria.

9. Clarify whether the minimum intended follow-up was three months for every participant or whether follow-up ended earlier when healing occurred.

10. The statement that the Kaplan–Meier curve presents the data “as a hazard rate” appears technically inaccurate. A standard Kaplan–Meier curve estimates the survival or event-free probability over time, rather than directly presenting the hazard rate. This wording should be corrected.

11. Clarify how censoring was handled for participants who were transferred, withdrawn from clinic, lost to follow-up or unhealed at study completion.

12. The authors should state whether wound healing assessors were trained or whether any standardisation procedures were used across clinics.

13. The potential measurement error introduced by the simple ruler method should be presented as a methodological limitation rather than mainly as an explanation for variation in wound area.

14. Clarify whether compression therapy was recorded as a binary variable or whether type, pressure, adherence and changes over time were documented.

15. The manuscript should use consistent terminology for the Biobank. It is referred to as both “ComplexWounds@Manchester – Biobank” and “Wounds@Manchester Biobank.”

16. Clarify whether the reported cost of £65.53 per sample included laboratory biomarker assays or only collection, transportation, processing and storage.

17. The costs should be presented as study-specific estimates rather than general estimates applicable to other settings.

18. The statement that there was “no disruption” to clinic procedures should be supported by an explanation of how disruption was assessed. It appears to have been based on observation rather than a formal measure.

19. The discussion should distinguish feasibility, acceptability, fidelity and scalability. These concepts should not be used interchangeably.

20. The authors should report whether any dressing samples were unusable, contaminated, delayed, insufficient in volume or excluded from processing.

21. The conclusion should not state that prognostic-factor changes can be captured unless the manuscript demonstrates that the relevant prognostic variables and biomarkers were measured successfully over time.

22. The strengths and limitations section should explicitly acknowledge that no formal sample size calculation or prespecified progression criteria were used.

23. The term “large cohort size” in the article summary should be reconsidered. A sample of 100 may be reasonable for a feasibility study, but it is not necessarily large for prognostic-factor or prognostic-model research.

**Reviewer**	**4**
**Name**	**Team, Victoria**
**Affiliation**	**Monash University, School of Nursing and Midwifery**
**Date**	**22-Jul-2026**
**COI**	**None**

Dear colleagues

I read your manuscript with interest. Your study is well conducted; and the findings are appropriately presented. I have only minor suggestions.

My main suggestion is to expand your literature review, citing relevant works of the UK and global researchers. I have included a few references by Sebastian Probst, Georgina Gethin, Peter Franks, and Carolina Weller for your consideration. You may find these studies helpful in justifying the need for your research. In particular, you could discuss the demographic and clinical profile of patients with venous leg ulcers (VLUs), their quality of life and health literacy levels, as well as the low rate of compression therapy prescribing in primary care settings. The role of population ageing, the rising prevalence of chronic illnesses, and the increasing burden of complex chronic wounds could also be discussed to further justify the significance and timeliness of the study.

Probst, S., Saini, C., Gschwind, G., Stefanelli, A., Bobbink, P., Pugliese, M. T., Cekic, S., Pastor, D., & Gethin, G. (2023). Prevalence and incidence of venous leg ulcers-A systematic review and meta-analysis. International wound journal, 20(9), 3906–3921. https://doi.org/10.1111/iwj.14272

Gethin, G., Vellinga, A., Tawfick, W., O'Loughlin, A., McIntosh, C., Mac Gilchrist, C., Murphy, L., Ejiugwo, M., O'Regan, M., Cameron, A., & Ivory, J. D. (2021). The profile of patients with venous leg ulcers: A systematic review and global perspective. Journal of tissue viability, 30(1), 78–88. https://doi.org/10.1016/j.jtv.2020.08.003

Bouguettaya, A., Gethin, G., Probst, S., Sixsmith, J., Team, V., & Weller, C. (2023). How health literacy relates to venous leg ulcer healing: A scoping review. PloS one, 18(1), e0279368. https://doi.org/10.1371/journal.pone.0279368

Liu, S., Team, V., Qiu, Y., & Weller, C. D. (2022). Investigating quality of life instrument measurement properties for adults with active venous leg ulcers: A systematic review. Wound repair and regeneration : official publication of the Wound Healing Society [and] the European Tissue Repair Society, 30(4), 468–486. https://doi.org/10.1111/wrr.13034

Franks, P. J., Barker, J., Collier, M., Gethin, G., Haesler, E., Jawien, A., Laeuchli, S., Mosti, G., Probst, S., & Weller, C. (2016). Management of Patients With Venous Leg Ulcers: Challenges and Current Best Practice. Journal of wound care, 25 Suppl 6, S1–S67. https://doi.org/10.12968/jowc.2016.25.Sup6.S1

Weller, C. D., Richards, C., Turnour, L., & Team, V. (2021). Venous leg ulcer management in Australian primary care: Patient and clinician perspectives. International journal of nursing studies, 113, 103774. https://doi.org/10.1016/j.ijnurstu.2020.103774

Weller, C. D., Richards, C., Turnour, L., Patey, A. M., Russell, G., & Team, V. (2020). Barriers and enablers to the use of venous leg ulcer clinical practice guidelines in Australian primary care: A qualitative study using the theoretical domains framework. International journal of nursing studies, 103, 103503. https://doi.org/10.1016/j.ijnurstu.2019.103503

Weller CD, Bouguettaya A, Britt H, Harrison C. Management of people with venous leg ulcers by Australian general practitioners: An analysis of the national patient-encounter data. Wound Rep Reg. 2020; 28: 553–560. https://doi.org/10.1111/wrr.12820

My other minor suggestion is to fix unformatted citations appearing in the study protocol uploaded as a supplementary file.

VERSION 1 - AUTHOR RESPONSE

<u>Reviewer comment</u>	<u>Author responses and changes made</u>
**Clarify study identity, design and scope:**<br><br>Reviewer 1: Although the study is presented as a prognostic biomarker study, no biomarker data are reported. In reality, this manuscript evaluates the feasibility of collecting biological samples rather than investigating biomarkers themselves. The title and abstract should reflect this more clearly to avoid misleading readers.	We thank the Editor and reviewers for these helpful comments. We have addressed these points and revised the manuscript throughout to clarify that the current paper reports a feasibility cohort study, rather than an analysis of prognostic factors. Specifically, we have revised the title, abstract objective, article summary, study design description, methods, results, discussion and conclusion to ensure consistent terminology and to avoid implying that prognostic-factor associations were evaluated in the current

Reviewer 2: Please use the term feasibility cohort study throughout rather than interchanging it with pilot study - no associations have been drawn.	study. We now describe the study consistently as a feasibility cohort study and have clarified that recruitment, retention, clinical data collection, follow-up procedures and repeated biological sampling were assessed for feasibility. We have also distinguished the feasibility outcomes assessed in this study from the descriptive clinical outcomes collected to test measurement procedures, and from the prognostic analyses planned for a future definitive study.
Reviewer 3: However, several conceptual and reporting issues need to be addressed before the manuscript can be considered scientifically clear and internally consistent. My main concern relates to the mismatch between the title, stated purpose, outcome measures, results and conclusions. The manuscript is presented in several places as though it investigated prognostic factors for venous leg ulcer healing. However, the study was primarily designed to assess the feasibility of conducting a future prognostic cohort study. No prognostic-factor associations, prognostic models, or validation analyses are presented. The manuscript should therefore be revised so that its identity as a feasibility study is maintained consistently throughout. The title, objective, outcome measures, abstract results, conclusion, article summary and discussion should clearly distinguish between:<br><br>• The feasibility outcomes assessed in the current study;<br><br>• The descriptive clinical outcomes collected to test whether they could be measured; and<br><br>• The prognostic analyses intended for a future definitive study.	The title has now been revised to include the study design. The new title is: 'Feasibility of a prospective cohort study to support future investigation of prognostic factors for venous leg ulcer healing'<br><br>The term 'feasibility cohort study' has now been used throughout.<br><br>The abstract objective has been revised to ensure there is distinction between feasibility and future prognostic aim: 'Objectives: To evaluate the feasibility of recruitment, longitudinal clinical data collection and repeated dressing-sample collection within routine NHS community venous leg ulcer care, to inform a future prospective cohort study evaluating prognostic factors for venous leg ulcer healing.'
Reviewer 3: 1. Mismatch between the title and the actual study purpose<br><br>The title is:<br><br>Feasibility of a prospective cohort study to investigate prognostic factors for venous leg ulcer healing.<br><br>This wording may give the impression that prognostic factors were investigated in the current study. However, the Methods section states that the study was designed to assess the feasibility of running a larger cohort study that would subsequently explore prognostication.<br><br>The current study therefore assessed the feasibility of conducting future prognostic research and not investigating prognostic factors directly. No associations between candidate prognostic factors and healing outcomes are analysed, and no prognostic model is developed or validated.	In addition, the Study design and ethics section of the manuscript has now been revised for clarity: 'The study was designed as a prospective, longitudinal feasibility cohort study to assess whether procedures needed for a future prognostic-factor study could be delivered within routine NHS community venous leg ulcer clinics. The study aimed to explore the feasibility of patient recruitment, clinical data access and dressing sample collection with variable follow-up (minimum follow-up time of 3 months or until reference ulcer healed) within NHS clinics in Manchester, while minimising disruption, assessing variation in clinical practices and exploring the ability to record outcomes in routine practice.'

The authors should revise the title to reflect the actual study purpose more accurately. The title should avoid implying that prognostic factors were evaluated unless such analyses are included.<br><br>Reviewer 3: 2. The abstract objective combines the current feasibility aim with the future prognostic aim<br><br>The abstract objective states:<br><br>“To assess the feasibility of conducting a robust prospective cohort study embedded within routine NHS community care to identify prognostic factors for venous leg ulcer healing.”<br><br>This sentence combines the immediate purpose of the current study with the intended purpose of a future study. Consequently, it is unclear whether the current study aimed to assess feasibility, identify prognostic factors, or both.<br><br>The objective should distinguish these two levels explicitly. For example:<br><br>To assess the feasibility of conducting a prospective cohort study, embedded within routine NHS community care, that could subsequently evaluate prognostic factors for venous leg ulcer healing.<br><br>Alternatively:<br><br>To evaluate the feasibility of recruitment, longitudinal clinical data collection and repeated biological sampling for a future prospective study of prognostic factors for venous leg ulcer healing.<br><br>This distinction should also be maintained consistently in the introduction, methods, discussion and conclusion.<br><br>Reviewer 3: The methods should distinguish study design from feasibility purpose<br><br>The manuscript alternates between describing the study as:<br><br>• A prospective longitudinal cohort study;<br><br>• An observational prospective cohort study; and<br><br>• A single-group repeated-measures feasibility study.<br><br>These descriptions are not necessarily incompatible, but the hierarchy should be clear. The study appears to	

be a prospective longitudinal feasibility cohort study with repeated measurements.<br><br>The authors should use one consistent description throughout the abstract, methods, article summary and discussion. Calling it simply an observational cohort study may overemphasise its prognostic design and understate its feasibility purpose.<br><br>Reviewer 3: The title should be revised so that it does not imply that prognostic factors were investigated in the current study.	
**Define feasibility criteria and progression thresholds:**<br><br>Reviewer 1: My main concern is that the manuscript does not clearly define what constituted a successful feasibility study before recruitment began. Although a number of feasibility questions are listed, there are no predefined progression criteria or thresholds for recruitment, retention, sample collection, or data completeness. As a result, the conclusion that the study was feasible is based largely on the authors' interpretation rather than objective criteria. This would be strengthened by referencing established guidance for feasibility studies and stating what outcomes would have been considered acceptable.<br><br>Reviewer 3: The study lacks clearly prespecified feasibility criteria or progression thresholds<br><br>The manuscript lists several feasibility questions, but it does not appear to provide prespecified criteria for determining whether the study would be considered feasible. For example, no thresholds are presented for:<br><br>• An acceptable recruitment rate;<br><br>• An acceptable consent rate;<br><br>• Minimum retention;<br><br>• Acceptable levels of missing data;<br><br>• Minimum number of dressing samples;<br><br>• Acceptable transport and processing time;<br><br>• Acceptable cost;<br><br>• Acceptable disruption to routine clinic procedures; or	We thank the reviewers for these thoughtful comments, which have prompted us to reflect further on, and clarify, the aims and approach of this work.<br><br>In this study, we were considering feasibility in a relatively broad and early-stage sense: whether this approach to prognosis research linked to wound healing outcomes could be undertaken at scale, and what undertaking it would involve. The study was not designed as a feasibility study for a specific, predefined definitive study. Consequently, we did not set formal progression criteria of the kind that might be used to determine whether a particular future trial or evaluation should proceed.<br><br>Instead, our aim was to characterise key areas of uncertainty that would inform the design of subsequent research. Our questions were therefore exploratory and empirical: for example, what recruitment rate can be achieved using this approach in this setting? What proportion of participants is retained? How complete are the data, and where does missingness arise? How acceptable and practicable are the study processes? Because of the dearth of research of this type in this field that has been undertaken appropriately and the lack of practical insights allowing exploration of feasible approaches – this early exploration was required.<br><br>This distinction is important because, in the absence of a predefined subsequent study, there was no clear basis on which to specify that a particular recruitment, retention or data-completeness threshold would represent 'success' or justify progression. The

• The proportion of participants with complete healing ascertainment.<br><br>Without prespecified progression criteria, the conclusion that the study was feasible is based largely on post hoc interpretation.<br><br>The authors should clarify whether formal feasibility criteria were specified in the protocol or registration. If they were, these should be reported and the findings evaluated against them. If they were not, this should be acknowledged as a limitation. The authors should avoid presenting feasibility as an entirely objective conclusion when the decision criteria were not defined in advance.	acceptability of these parameters will depend partly on the design, scale and objectives of any subsequent study. Our intention was therefore to generate the information needed to make those judgements and to inform the design of future studies including potential sample sizes, rather than to make a binary go/no-go decision about progression to a particular study.<br><br>We recognise that this rationale was not sufficiently explicit in the manuscript and have revised the text to clarify the purpose of the feasibility study and the way in which feasibility was assessed.<br><br>This is an area where robust prospective research is very limited (we have recently had a prognostic factor review for VLU healing accepted for publication in Wound Repair and Regeneration that shows very limited robust data) and we really do believe that the insights from this feasibility work are important for the field in exploring what could be achieved.
**Separate feasibility outcomes from descriptive clinical findings:**<br><br>Reviewer 1: The manuscript would also benefit from a clearer separation between feasibility outcomes and descriptive clinical findings. Results such as healing rates, infection rates, and median healing time are interesting, but they are secondary observations and should not be interpreted as evidence of feasibility. The emphasis should remain on whether recruitment, follow-up, data collection, and repeated sampling were achievable within routine community practice.<br><br>Reviewer 3: Feasibility outcomes are not clearly separated from descriptive clinical outcomes<br><br>The abstract lists the following outcome measures:<br><br>“Participant recruitment, participation, clinical activity, time to healing estimation, dressing sample collection and participant retention.”<br><br>Some of these are clearly feasibility outcomes, including recruitment, participation, sample collection and retention. However, “clinical activity” is vague, and “time to healing estimation” is primarily a clinical	We have revised the manuscript to clearly distinguish feasibility outcomes from descriptive clinical findings throughout the Abstract, Methods and Results. Feasibility outcomes are now prioritised and organised around recruitment, participation, operational feasibility, data completeness, biological sampling, retention and follow-up. Median time from recruitment to documented healing data has now been removed as this was not a primary outcome for this feasibility study. Descriptive clinical findings, including healing, infection and dressing use have been reframed as exploratory observations used to assess the feasibility of collecting these measures in a future study. We have also revised the abstract to reduce emphasis on clinical findings and to focus more clearly on outcomes directly related to the study’s feasibility objectives. Figures and tables have been reorganised to allow the primary outcome results to be included in the main manuscript rather than supplementary material.

outcome unless it is explicitly framed as an assessment of whether healing data could be collected adequately for a future study.<br><br>The Methods section provides a more extensive list of feasibility questions, but these are not organised into clearly defined primary and secondary feasibility outcomes. The authors should distinguish between:<br><br>• Recruitment and participation feasibility: eligibility, consent, recruitment rate, recruitment time and translation requirements.<br><br>• Operational feasibility: integration into clinic workflow, disruption to routine care, staff requirements, data access and Biobank support.<br><br>• Data-collection feasibility: completeness of baseline and follow-up data, ability to record compression use, infection, wound area and healing.<br><br>• Biological-sampling feasibility: number and frequency of dressing samples, transport time, processing, storage and cost.<br><br>• Retention and follow-up feasibility: attrition, reasons for exit, duration of follow-up and completeness of healing ascertainment.<br><br>• Descriptive clinical outcomes: time to healing, healing proportion, infection frequency, dressing types and compression use.<br><br>Clinical findings can be reported, but they should be clearly described as exploratory or descriptive outcomes collected to assess measurement feasibility rather than as substantive prognostic findings.<br><br><br>Reviewer 3: The abstract results overemphasise descriptive clinical findings<br><br>The Results section of the abstract gives substantial prominence to median time to healing, the proportion healed, baseline wound infection, the organisms identified and the number of dressing types used.<br><br>Although these findings are informative, the abstract should prioritise the outcomes that directly answer the feasibility objective. These include:<br><br>• The number and proportion of eligible participants recruited;	

• The time required for screening, consent and baseline assessment;<br><br>• Participants retention and reasons for study exit;<br><br>• The completeness of clinical data;<br><br>• The number of repeated dressing samples successfully collected;<br><br>• Transport and processing time;<br><br>• Operational costs;<br><br>• The ability to integrate the procedures into routine clinics; and<br><br>• Whether routine care was disrupted.<br><br>The finding that Pseudomonas aeruginosa was the most commonly identified organism among a very small number of infected participants is not central to the principal feasibility question. Given the limited abstract word count, it could be removed or moved to the main Results section.<br><br>Similarly, the finding that 45 different dressing types were used is relevant mainly because dressing heterogeneity may affect the future use of wound-fluid biomarkers. This relationship should be made explicit rather than presenting dressing heterogeneity as a standalone clinical result.	
**Clarify interpretation of time from recruitment to healing:**<br><br>Reviewer 1: There are also some issues with the statistical reporting. The Kaplan-Meier curve estimates the probability of healing over time rather than the hazard rate, and this terminology should be corrected throughout the manuscript. It would also be helpful to provide confidence intervals for the main descriptive outcomes, particularly recruitment, healing, and infection rates<br><br>Reviewer 1: The reported median healing time should also be interpreted with caution, as participants entered the study at different stages of ulcer duration. Measuring healing from the time of recruitment rather than ulcer onset is likely to underestimate the true healing time for many patients, and this limitation deserves greater emphasis in the discussion.	Thank you for your comments, the time from recruitment to healing data including the Kaplan-Meier curve has now been removed from this manuscript as this was not a primary outcome for this feasibility study. Confidence intervals have now been included.

Reviewer 3: The statement that the Kaplan–Meier curve presents the data “as a hazard rate” appears technically inaccurate. A standard Kaplan–Meier curve estimates the survival or event-free probability over time, rather than directly presenting the hazard rate. This wording should be corrected.<br><br>Reviewer 3: Clarify how censoring was handled for participants who were transferred, withdrawn from clinic, lost to follow-up or unhealed at study completion.<br><br>Reviewer 3: Time to healing should be interpreted cautiously because recruitment did not begin at ulcer onset<br><br>The manuscript reports a median time to healing of 10 weeks from recruitment. However, participants were recruited at different stages of ulcer development and healing, with substantial variation in wound duration at baseline. Therefore, this estimate is not the total time from ulcer onset to healing.<br><br>This limitation is acknowledged in parts of the manuscript, but it should be made more explicit wherever the healing estimate is presented, especially in the abstract. The preferred wording would be:<br><br>Median time from study recruitment to documented healing was 10 weeks.<br><br>The authors should avoid wording that could be interpreted as the natural healing duration of venous leg ulcers. Recruitment closer to ulcer onset and restriction or stratification by ulcer duration may be needed in a future prognostic study.<br><br>Reviewer 3: Report “time from recruitment to healing” rather than “time to healing” throughout.	

**Discuss generalisability and selection bias:**<br><br>Reviewer 1: The generalisability of the findings should be discussed in more detail. Recruitment was limited to three clinics within a single NHS Trust, and patients receiving home-based care and non-English speakers were excluded. These factors may introduce selection bias and limit the applicability of the findings to other healthcare settings. Similarly, the very high consent rate is impressive but may not be reproducible in a larger multicentre study.	The generalisability of the findings have now been discussed in more detail in the Discussion section: 'The generalisability of these findings should be interpreted in the context of the study design and recruitment setting. Participants were recruited from three community leg ulcer clinics within a single NHS Trust, and therefore may not fully represent patients managed in other geographical areas, service models or healthcare systems. In addition, patients receiving home-based care and those unable to understand English were not eligible to participate, which may have introduced selection bias by excluding individuals with different clinical needs, levels of mobility, social support or communication requirements. These factors limit the extent to which the feasibility findings can be applied to the wider population of people with venous leg ulcers. Although the high consent rate suggests that the study procedures were acceptable to eligible participants, this finding should be interpreted cautiously, as it may not be replicated in a larger multicentre study involving more diverse clinical settings and participant populations.'
**Clarify cost analysis methods and scope:**<br><br>Reviewer 1: Finally, the cost analysis is useful but would benefit from additional detail. It would be helpful to describe how staff costs, transport, laboratory consumables, and overheads were calculated, as this information would assist other groups planning similar studies.<br><br>Reviewer 3: Clarify whether the reported cost of £65.53 per sample included laboratory biomarker assays or only collection, transportation, processing and storage.<br><br>Reviewer 3: The costs should be presented as study-specific estimates rather than general estimates applicable to other settings.	Information on how the costs were calculated has now been included in the Methods data analysis section: 'Study costs included Biobank charges, staff time, transport, storage and laboratory consumables, with total consumables and Biobank costs calculated by dividing by the number of samples collected and participants recruited, respectively, to estimate average per-sample and per-participant costs.'<br><br>Clarification of the cost has also been explained in more detail including emphasis that this was study-specific estimates in the Results section: 'The study-specific cost per sample resource use is outlined in Table 4. In this feasibility study, the cost per sample was calculated as £65.53 and included collection, transportation, processing and storage only; laboratory biomarker assays were not included in this estimate. The study-specific cost to recruit a participant via the Biobank was calculated as £157.10 per participant. The total study cost to recruit 100 participants

	(£15,710.00) and process 503 samples (£32,959.99) was £48,669.99.'
**Clarify confirmation of venous aetiology and absence of arterial disease:**<br><br>Reviewer 2: For those in whom ABPI is not possible how was absence of arterial disease confirmed?	Confirmation of venous aetiology has now been explained more clearly in the eligibility section of the Methods: 'Ankle brachial pressure index (ABPI) was > 0.8. For those in whom ABPI was not possible, Toe brachial pressure index (TBPI) >60mmHG was used as an alternative to confirm absence of arterial disease. If unable to obtain both ABPI/TBPI, referral to a specialist was made.'
**Primary feasibility question:**<br><br>Reviewer 2: Please state the primary feasibility q first in the aims.<br><br>Reviewer 3: In the abstract objective, separate the immediate feasibility aim from the future prognostic aim.	The feasibility question and aim have been revised in the abstract to reflect these comments: 'Objectives: To evaluate the feasibility of recruitment, longitudinal clinical data collection and repeated dressing-sample collection within routine NHS community venous leg ulcer care, to inform a future prospective cohort study evaluating prognostic factors for venous leg ulcer healing.
**Clarify eligibility, ineligibility and consent denominator:**<br><br>Reviewer 2: There were a lot of people ineligible because they did not have an ulcer? Was this not an essential criteria?<br><br>Reviewer 3: Exclusion of non-English-speaking participants needs stronger consideration<br><br>Non-English-speaking patients were excluded, and one potentially eligible patient with translation needs was identified. Although the number was small in this sample, the exclusion criterion has implications for inclusiveness and generalisability.<br><br>A future definitive study should consider translated participant materials and interpreter support. The authors should discuss whether language-related exclusion could become more important when recruitment expands to more ethnically and linguistically diverse areas.<br><br>The study's very high consent rate should also be interpreted in the context of this eligibility restriction.	Ulcer presence was an essential eligibility criterion for the study. However, patients may continue to attend the clinic after their ulcers have healed, for example for ongoing compression therapy and follow-up care. As a result, a substantial number of patients screened in the clinic population did not have an active ulcer at the time of assessment, which accounts for the 845 patients recorded as having "no ulcer." A sentence has been included in the Methods section under data and sample collection heading to address this: 'Ulcer presence was an essential eligibility criterion for the study, but because patients may continue attending clinic after healing for compression therapy and follow-up care, many screened patients did not have an active ulcer at assessment.'<br><br>The exclusion of Non-English speaking participants has now been discussed and acknowledged in more detail in the Discussion section: 'The English-language eligibility criterion was pragmatic for this feasibility study but may have limited participation among people who speak languages other than English, with

Reviewer 3: Clarify whether the reported 99% consent rate refers only to eligible patients who were approached.<br><br>Reviewer 3: Explain why the absence of an exudating wound was treated as a reason for ineligibility when this criterion is not clearly included in the listed eligibility criteria.	implications for inclusivity and generalisability. This may be particularly relevant if recruitment is extended to more ethnically and linguistically diverse areas, where English-only materials could reduce equitable access and affect representativeness. Future studies should consider translated participant materials and interpreter support during recruitment, consent and follow-up. The high consent rate in this study should also be interpreted in this context, as eligible participants may have faced fewer communication barriers than would be expected in more linguistically diverse settings.'<br><br>Patients who met the eligibility criteria were approached and invited to provide consent to participate. Of these, 99% consented, while one person declined to take part.<br><br>This has now been changed to explicitly state that the reason for ineligibility was 'the absence of an open wound'.
**PPIE group:**<br><br>Reviewer 2: PPI group could expand to include a caregiver or spouse.	A consideration for expanding the PPIE group in the future has now been included in the Discussion section: 'The lived experience advisory group established alongside this feasibility study included individuals with direct experience of venous leg ulcers. Future work would benefit from expanding this group to include caregivers or spouses, to capture additional perspectives on supporting someone with a venous leg ulcer.'
**Consider research nurse support for future studies:**<br><br>Reviewer 2: It is creditable that the patients were recruited bearing in mind the extra time per NHS nurse. Could NIHR primary care nurses help in future trials?	The additional time required from NHS clinic nurses is an important consideration for future studies. In this feasibility study, recruitment and study procedures were supported by the research team and Biobank staff alongside routine clinic staff. Thus, there was little burden on the NHS nurses as the research procedures did not impact on their routine caseloads. However, involvement of NIHR primary care research

	nurses along with relevant funding could provide valuable additional capacity in a future definitive trial.
**Address wound measurement method and limitations:**<br><br>Reviewer 2: Wound measurement using a ruler is rather basic. Suggest use ImitoMeasure (WoundGenius) to record and measure area and characteristics in future studies. This also allows independent endpoint verification.	While we agree that a more objective method of measuring these wounds would be optimal, we wanted to preserve standard practice in the community clinics. The ruler method is how nurses in these clinics currently measure wounds, so it was important to maintain consistency.
Reviewer 3: The potential measurement error introduced by the simple ruler method should be presented as a methodological limitation rather than mainly as an explanation for variation in wound area.	The use of a simple ruler method may introduce measurement error and this has been acknowledged as a methodological limitation. We have therefore included text in the Discussion section to present this issue more clearly as a limitation, while noting that this approach reflected standard practice in the community clinics where measurements were taken.<br>Discussion: 'A limitation relates to the use of the ruler method to measure wound area, which may have introduced measurement error. Future studies could incorporate digital wound imaging and use of platforms such as ImitoMeasure (WoundGenius) to record wound area and characteristics. This approach would also support independent endpoint verification.41 However, the ruler approach reflected standard practice in the community clinics where measurements were taken and helped maintain consistency with routine clinical assessment.'
**Clarify wound healing assessment and standardisation:**<br><br>Reviewer 2: Is one clinician at one timepoint enough to adjudicate wound healing?<br><br>Reviewer 3: The authors should state whether wound healing assessors were trained or whether any standardisation procedures were used across clinics.	In the community leg ulcer clinics included in this feasibility study, wound healing was assessed by the one clinician/nurse at the routine clinic visit. Clinicians/nurses working in these services are experienced in leg ulcer assessment and management, and this approach reflected standard practice within these community clinics. As the aim of the study was to assess feasibility in a real-world community clinic setting, the assessment process was intentionally aligned with usual care rather than introducing additional adjudication or formal

	standardisation procedures beyond those normally used in clinical practice.<br><br>This has now been included in the Discussion section of the manuscript: 'Similarly, wound healing was assessed by the leg ulcer clinic nurse responsible for each participant's routine visit. These nurses were experienced in leg ulcer assessment and management, and this approach was consistent with the study's aim of evaluating feasibility in a real-world community clinic setting, rather than introducing additional adjudication or formal standardisation procedures beyond usual care.'
**Clarify Pseudomonas treatment:**<br><br>Reviewer 2: How was the pseudomonas treated?	In the leg ulcer clinic, suspected or confirmed Pseudomonas colonisation or infection was managed with good wound hygiene, including regular cleansing and debridement where appropriate, alongside antimicrobial dressings such as iodine, silver, or honey-based products. Systemic antibiotics were reserved for ulcers showing clinical signs of spreading or systemic infection, or where the wound failed to improve, and were guided by culture and sensitivity results.
**Abstract - replace subjective descriptors with numerical results:**<br><br>Reviewer 3: "Minimal attrition" and "missing data were negligible" should be replaced with numerical reporting<br><br>The abstract states that retention was high, attrition was minimal and missing data were negligible. These terms are subjective and are not supported by prespecified thresholds.<br><br>The authors should report the relevant numbers directly. For example:<br><br>• 4 participants were lost to follow-up;<br><br>• 3 were withdrawn from the study;<br><br>• 9 were withdrawn from the clinic;<br><br>• 10 remained unhealed at the end of follow-up;<br><br>• No participants specifically requested withdrawal after recruitment;	The terms 'high retention', 'minimal attrition' and 'negligible missing data' have now been removed or replaced with numerical data. The Abstract results section now reads: 'Of 101 eligible patients, 99% consented, recruiting 100 participants and no participants requested withdrawal after recruitment. We were able to collect baseline and follow-up data, compression use, infection, wound area and healing outcomes data from the majority of participants using this approach with 74 participants achieving complete healing. Of the 26 participants who did not heal during follow-up, 10 remained unhealed at study end, 4 were lost to follow-up, 9 were withdrawn from clinic and 3 were withdrawn from the study due to change in ulcer aetiology; no participants requested withdrawal after recruitment. It was reassuring that were able to collect all of this follow-up data until the required endpoint and this supports the feasibility of collecting these

• Healing outcome data were complete;<br>• BMI was missing for 11% of participants; and<br>• Missing for most other variables was low.<br>This would provide a more transparent account than the terms "minimal" or "negligible." It is also important to distinguish active participant withdrawal from other forms of study exit.<br><br>Reviewer 3: Replace subjective terms such as "high retention," "minimal attrition" and "negligible missing data" with numerical results or prespecified criteria.	data successfully in a future study. The median time taken from collection of the last dressing sample, transport and processing before storage at the laboratory for all clinic sessions was 85 minutes. Healing outcome data were complete, BMI was missing for 11 participants, and missing data for most other baseline and follow-up variables were 0–2%. Biological sampling was feasible, although dressing practices were heterogeneous, with 45 dressing types used and a mean of five samples were collected per participant; this should be considered in future studies that aim to collect wound-fluid biomarkers. Integration of these procedures, with Biobank support, into clinic workflow was successful and did not disrupt routine care based on researcher observation and informal feedback from clinic staff.'
**Define retention and distinguish reasons for study exit:**<br>Reviewer 3: Retention is not clearly defined and may be conflated with healing-related study exit<br>The manuscript reports high retention, but 74% of participants exited after healing, while the remaining participants exited for several different reasons. In a wound-healing study, successful healing is an expected endpoint rather than evidence of retention in the conventional sense.<br>The authors should define retention carefully. Retention could refer to the proportion of participants who remained under observation until one of the following:<br>• Confirmed healing;<br>• Planned end of follow-up; or<br>• A valid documented clinical exit.<br>Participants lost to follow-up should be distinguished from participants who exited because of healing, transfer of care, discharge from clinic or study completion.<br><br>The reported median participation duration of eight weeks should also not be interpreted automatically as evidence of high retention because follow-up duration was partly determined by healing time and recruitment stage. A participant recruited near the end of the healing	The data analysis section in the manuscript has been revised to define retention as the proportion of participants who remained under observation in the study until confirmed healing. Participants who did not remain under observation until confirmed healing have been distinguished from those who completed follow-up through healing in Table 5, to avoid conflating successful healing-related study exit with loss to follow-up.<br>Data analysis: 'Participant retention is defined as the proportion of participants who remained under observation in the study until confirmed healing and is displayed as counts and percentages.'<br><br>The link between retention and participant duration has now been removed from the manuscript.

trajectory may have a short study duration despite being fully retained.	
**Revise conclusion to reflect findings of the current study:**<br><br>Reviewer 3: The conclusion should focus on what the study directly demonstrated<br><br>The abstract conclusion begins:<br><br>"Identifying prognostic factors could improve individualised treatment and management..."<br><br>This is background justification rather than a conclusion derived from the study. The current study did not identify prognostic factors or demonstrate improved personalised management.<br><br>The conclusion should begin with the feasibility findings. For example:<br><br>Biobank-supported recruitment, longitudinal clinical data collection and repeated dressing-sample collection could be embedded within routine community venous leg ulcer clinics without apparent disruption to usual care. The study provides operational information for a larger prospective prognostic cohort, although longer follow-up, recruitment closer to ulcer onset and prespecified progression criteria will be required.<br><br>The final claim that the study establishes a model for identifying prognostic biomarkers should also be moderated. The study demonstrates the feasibility of the collection infrastructure, but not that the collected samples will necessarily be analytically valid across heterogeneous dressings or clinically useful for prognosis.<br><br>Reviewer 3: The conclusion should not state that prognostic-factor changes can be captured unless the manuscript demonstrates that the relevant prognostic variables and biomarkers were measured successfully over time.	The Abstract Conclusion has now been revised to focus on the feasibility findings and the final claim has been moderated: 'Biobank-supported recruitment, longitudinal clinical data collection and repeated dressing-sample collection were integrated into routine community venous leg ulcer clinics with no observed impact on usual care. These findings support feasibility of larger-scale prospective cohort studies evaluating prognostic factors for venous leg ulcer healing, although future studies should include longer follow-up, recruitment closer to ulcer onset and prespecified progression criteria.'<br><br>The final conclusion has been revised to state the following: 'Infrastructure has successfully been set up in three NHS community leg ulcer clinics to produce an efficient workflow from recruitment through to dressing collection, transportation and sample extraction. Recruitment and retention rates were high and there were no disruptions to the normal clinic procedures and clinic times. Issues with dressing sample collection and transportation were minimal and scale-up in recruitment and sample collection is feasible using the current methods. The recruitment and collection workflow set up here should facilitate long term repeated sampling and allow expansion of recruitment centres for larger studies in this field. Overall, this feasibility study suggests that a future prospective cohort study evaluating prognostic factors for venous leg ulcer healing could be delivered within routine community care, although further investment and refinement would be required. Future large-scale studies should include longer follow-up, recruitment closer to ulcer onset and prespecified progression criteria.'

<table>
<tr><td></td><td></td></tr>
<tr><td>Revise strengths and limitations to reflect feasibility design:

Reviewer 3: The article summary does not adequately reflect the feasibility design

The first strength states that the observational prospective cohort design is ideal for identifying and validating prognostic factors. This describes the rationale for the future definitive study rather than a strength of the current feasibility study.

The strengths and limitations should instead focus on what was tested in the present study. Possible strengths include:

• Recruitment through routine community clinics;

• High consent among eligible participants;

• Repeated clinical and biological sample collection;

• Use of established Biobank infrastructure;

• Recording of operational time and resource costs;

• minimal apparent disruption to routine care; and

• complete ascertainment of healing status.

Important limitations include:

• Absence of prespecified feasibility thresholds;

• Recruitment at different stages of wound healing;

• Variable and sometimes short follow-up;

• Exclusion of non-English-speaking patients;

• Exclusion of patients receiving home-based care;

• Recruitment from only three clinics in one geographical area;

• Potential dependence on specialised Biobank infrastructure;

• Underrepresentation of ethnic minority groups;

• Use of routine ruler-based wound measurement;

• Dressing heterogeneity; and

• Limited assessment of recurrence.</td><td>The strengths and limitations summary section has now been revised to reflect these suggestions:

• This study design was to assess the feasibility of a prospective cohort study, as this approach is ideal for the identification and validation of prognostic factors in real-world populations.

• Research infrastructure is limited in community care settings; therefore, this study utilised the ComplexWounds@Manchester Biobank infrastructure to facilitate clinical data and biological sample collection enabling long term repeated sampling and allowing expansion for larger studies in this field.

• Monitoring recurrence rates and recruiting participants closer to ulcer inception to capture the full healing process would be desirable in the future.

• Whilst we explored the feasibility of undertaking research using the biobank facilitated approach, further exploration of the feasibility of specific prognostic studies, with appropriate sample sizes (informed by this study) along with prespecified progression criteria are required.

• Generalisability may be limited by selection bias, language and care-setting exclusions, ethnic underrepresentation, and single-area recruitment.</td></tr>
</table>

The article summary should be revised to distinguish current study achievements from future prognostic ambitions.<br><br>Reviewer 3: The strengths and limitations section should explicitly acknowledge that no formal sample size calculation or prespecified progression criteria were used.	
**Clarify scope of wound-fluid biomarker analysis:**<br><br>Reviewer 3: The role of wound-fluid biomarker analysis requires clearer explanation<br><br>The manuscript states that dressing samples were processed and wound fluid extracted and analysed for a separate biomarker study. However, the present manuscript does not report biomarker quality, concentration, assay performance or comparability across dressing types.<br><br>The authors should clarify the scope of the present paper. Did the feasibility assessment include only successful collection, transportation and storage, or did it also include evidence that the extracted wound fluid was analytically usable?<br><br>This is important because successful physical collection of a dressing sample does not necessarily demonstrate the feasibility of valid biomarker measurement. Different dressing materials may absorb, bind, dilute or alter proteins differently. Given that 45 dressing types were used, the authors should explain whether dressing type could affect protein recovery and whether this will require standardisation, adjustment or validation in the future definitive study.<br><br>If analytical validity is outside the scope of this paper, the authors should state this clearly and avoid implying that the complete biomarker pathway has been demonstrated to be feasible.<br><br>Reviewer 3: The authors should report whether any dressing samples were unusable, contaminated, delayed, insufficient in volume or excluded from processing.	The feasibility assessment in the present manuscript was limited to the practical aspects of collecting, transporting, storing and processing dressing samples, rather than the analytical validity of biomarker measurement. Detailed assessment of biomarker quality, assay performance, protein recovery and comparability across dressing types is outside the scope of this paper and will be addressed in related biomarker-focused publications. We have revised the language used in the manuscript to make this distinction clearer and to avoid implying that the full biomarker pathway has been demonstrated to be feasible. This point has also been outlined in the Data collection section in the Methods.<br><br>We have clarified the handling and processing of dressing samples in the manuscript. No dressing samples were delayed in processing. The only samples that yielded lower extraction volumes were from wounds that were close to healing, where reduced wound fluid on the dressing was expected. This has now been added to the manuscript results section under biological sampling for clarity.
**Discuss generalisability of the Biobank-supported model:**	

Reviewer 3: The generalisability of the Biobank-supported model should be discussed more critically<br><br>The manuscript concludes that this model could support expansion to additional centres. However, the feasibility observed here depended heavily on experienced Biobank research staff who attended clinics, collected data and samples, arranged transportation, stored records and coordinated laboratory processing.<br><br>This infrastructure may not be available in many community settings. The authors acknowledge this issue, but it should be treated as a central limitation rather than only a consideration for future scale-up.<br><br>The manuscript should distinguish between:<br><br>• Feasibility within a specialised Biobank-supported system; and<br><br>• Feasibility within routine community clinics without equivalent infrastructure.<br><br>The current findings primarily establish the former. Replication in settings with different staffing, transport distances, clinic structures and research capacity will be necessary before broader generalisability can be claimed.	We agree that the feasibility demonstrated in this study reflects implementation within a specialised Biobank-supported model, rather than feasibility within routine community clinics without equivalent infrastructure. We have revised the Discussion to make this distinction clearer and to present the reliance on experienced Biobank research staff and established sample-handling processes as a central limitation. We have also clarified that wider generalisability cannot be assumed without further evaluation in settings with different staffing models, transport arrangements, clinic structures and research capacity.<br><br>Discussion revisions: 'The feasibility demonstrated in this study should be interpreted within the context of the specialised Biobank-supported system. Recruitment, clinical data collection, dressing-sample collection, transport coordination and record management were supported by experienced Biobank research staff and established infrastructure. This support was central to successful delivery and may not be available in all routine community clinic settings. Therefore, the findings primarily demonstrate feasibility within a supported research infrastructure, rather than feasibility across community services more broadly. Further evaluation in centres with different staffing arrangements, transport distances, clinic workflows and research capacity will be needed before the model can be considered generalisable or scalable across wider community leg ulcer services.'
**Moderate claims about feasibility of future prognostic research:**<br><br>Reviewer 3: The discussion should avoid implying that prognostic research was successfully conducted<br><br>Several passages state that the study demonstrates that prognostic research can be carried out in the community. More precisely, the study demonstrates that procedures required for a future prognostic study—recruitment, longitudinal data collection and repeated sample collection—can be implemented in these clinics.<br><br>The authors should revise statements that imply that valid prognostic-factor research has already been completed. The present study did not evaluate candidate predictors, estimate their associations with	The Discussion section has been substantially revised to make clear that the study demonstrates the feasibility of procedures required to support a future prognostic study, including recruitment, longitudinal clinical data collection and repeated dressing-sample collection, rather than the feasibility or validity of prognostic-factor analysis itself.

healing, assess model performance or validate a prognostic model.<br><br>The distinction is important because feasibility of data and sample collection is necessary but not sufficient to establish feasibility of the full prognostic research programme.	
**Abstract - describe clinical outcomes more specifically:**<br><br>Reviewer 3: Replace "clinical activity" in the abstract outcome measures with a more specific description<br><br>Reviewer 3: Consider using the term "descriptive clinical outcomes" for healing, infection, wound characteristics, compression use and dressing type.	The outcome measures section in the abstract has been amended to specify clinical activity more clearly: 'Outcome measures: Participant recruitment, participation, feasibility of collecting baseline and follow-up clinical data, healing outcomes, dressing sample collection and participant retention.'
**Clarify intended follow-up period:**<br><br>Reviewer 3: Clarify whether the minimum intended follow-up was three months for every participant or whether follow-up ended earlier when healing occurred.	Minimum follow-up time was 3 months or until the reference ulcer healed and this has now been amended in the manuscript' Study design and ethics' section of the Methods.
**Clarify recording of compression therapy:**<br><br>Reviewer 3: Clarify whether compression therapy was recorded as a binary variable or whether type, pressure, adherence and changes over time were documented.	This has now been clarified in the Data and sample collection section of the Methods: 'Compression therapy was presented as a binary variable indicating whether compression was being received; however, the type of compression used was also documented.'
**Ensure consistent Biobank terminology:**<br><br>Reviewer 3: The manuscript should use consistent terminology for the Biobank. It is referred to as both "ComplexWounds@Manchester – Biobank" and Wounds@Manchester Biobank.	The terminology has been changed throughout the manuscript to name the biobank consistently as: "ComplexWounds@Manchester Biobank".
**Clarify assessment of disruption to clinics:**<br><br>Reviewer 3: The statement that there was "no disruption" to clinic procedures should be supported by an explanation of how disruption was assessed. It	Disruption to clinic procedures was not assessed using a formal measure; rather, this statement was based on researcher observation during recruitment

appears to have been based on observation rather than a formal measure.	and data collection, alongside informal feedback from clinic staff. We have revised the Discussion section in the manuscript to clarify this and to avoid implying that disruption was formally measured: 'A strength of this study was that the study procedures appeared to be feasible within routine NHS clinic workflows, with no major disruption observed during recruitment or data collection as data and sample collections were absorbed into waiting time and the clinic session itself. NHS Trusts outside of Greater Manchester may have different clinic times and structures, thus it would be essential to take this into account. Disruption was not assessed using a formal measure and this finding was based on researcher observation and informal feedback from clinic staff. Future studies should consider incorporating a more structured assessment of intervention burden and disruption to routine clinical practice.'
**Distinguish feasibility, acceptability, fidelity and scalability:**<br><br>Reviewer 3: The discussion should distinguish feasibility, acceptability, fidelity and scalability. These concepts should not be used interchangeably.	The Discussion has been revised to distinguish feasibility, acceptability, fidelity and scalability more clearly, and we have taken care not to use these concepts interchangeably. For example, feasibility is now discussed in relation to recruitment, retention and data collection processes; acceptability is discussed in terms of participant and staff engagement with the study procedures; fidelity is addressed in relation to whether the intervention and study procedures were delivered as intended; and scalability is considered separately in terms of the implications for delivering the approach in a larger definitive study.
**Revise wording about cohort size:**<br><br>Reviewer 3: The term "large cohort size" in the article summary should be reconsidered. A sample of 100 may be reasonable for a feasibility study, but it is not necessarily large for prognostic-factor or prognostic-model research.	The term 'large cohort size' has now been removed from the article summary.
**Expand literature review and contextual justification:**<br><br>Reviewer 4: My main suggestion is to expand your literature review, citing relevant works of the UK and	Thank you for this helpful suggestion. We have expanded the Introduction and included several of the

global researchers. I have included a few references by Sebastian Probst, Georgina Gethin, Peter Franks, and Carolina Weller for your consideration. You may find these studies helpful in justifying the need for your research. In particular, you could discuss the demographic and clinical profile of patients with venous leg ulcers (VLUs), their quality of life and health literacy levels, as well as the low rate of compression therapy prescribing in primary care settings. The role of population ageing, the rising prevalence of chronic illnesses, and the increasing burden of complex chronic wounds could also be discussed to further justify the significance and timeliness of the study.	suggested references. We have not included all of the suggested references, as some were based in the Australian healthcare context. While these studies are valuable, we felt they were less directly applicable to the present study given its UK primary care context, particularly in relation to service delivery, prescribing practices and healthcare system organisation.
**Correct unformatted citations in supplementary protocol:**<br><br>Reviewer 4: My other minor suggestion is to fix unformatted citations appearing in the study protocol uploaded as a supplementary file.	Unformatted citations have now been corrected in the supplementary Study protocol.

VERSION 2 - REVIEW

**Reviewer**	**1**
**Name**	**Aimanan, Karthigesu**
**Affiliation**	**Universiti Malaysia Sabah**
**Date**	**27-Aug-2026**
**COI**	

I think the manuscript has improved substantially after revision. The authors have addressed most of the major conceptual issues raised previously, particularly by clearly reframing the work as a prospective longitudinal feasibility cohort study, rather than suggesting that prognostic factors were actually evaluated.

I would now lean toward minor revision, with the following concise reviewer comments:

1. The manuscript has improved considerably following revision, and the distinction between the current feasibility study and the future prognostic-factor study is now much clearer. The revised title, objectives and study design appropriately reflect the actual scope of the work.

2. Please further streamline the Abstract Results section. It remains relatively lengthy and contains interpretative wording such as “It was reassuring that…”. I suggest presenting the key feasibility results objectively and numerically, particularly recruitment, retention, data completeness, sample collection and processing time, leaving interpretation to the Discussion.

3. The definition of retention could still be clarified. Seventy-four participants reached the expected endpoint of complete healing, while the remaining participants had several different reasons for study exit. These should continue to be clearly distinguished from true loss to follow-up, rather than being grouped conceptually as attrition. The authors have improved this substantially, but consistency throughout the manuscript should be checked.

4. The absence of prespecified progression criteria remains an important limitation. Since no predefined recruitment, retention, data-completeness or sampling thresholds determined whether progression to a larger study would be considered feasible, the conclusions regarding feasibility are necessarily partly interpretative. This should remain clearly acknowledged when discussing progression to a definitive study.

5. The claim regarding lack of disruption to routine clinical care should remain appropriately cautious. This was based on researcher observation and informal staff feedback rather than a formally measured outcome. The revised manuscript acknowledges this, and I recommend ensuring that wording such as “successful” does not inadvertently imply formally demonstrated acceptability or operational efficiency.

6. The generalisability of the findings should be emphasised. The study was conducted in only three community leg-ulcer clinics in Greater Manchester and benefited from established Biobank infrastructure. The feasibility demonstrated here may therefore not translate directly to centres without similar research infrastructure, staffing or wound-care pathways.

7. A final language and grammatical review is recommended. There are still several awkward sentences in the revised text, particularly in the Abstract (for example, “It was reassuring

that were able to collect…”). These can be corrected during final editing without altering the scientific content.

VERSION 2 - AUTHOR RESPONSE

**Reviewer 1 Comments:**	
1. The manuscript has improved considerably following revision, and the distinction between the current feasibility study and the future prognostic-factor study is now much clearer. The revised title, objectives and study design appropriately reflect the actual scope of the work.	Thank you for your comments.
2. Please further streamline the Abstract Results section. It remains relatively lengthy and contains interpretative wording such as “It was reassuring that…”. I suggest presenting the key feasibility results objectively and numerically, particularly recruitment, retention, data completeness, sample collection and processing time, leaving interpretation to the Discussion.	Any interpretive wording in the Abstract Results section has been removed, and only objective results are now presented:<br>‘Of 101 eligible patients, 99% consented, 100 participants were recruited over a period of 53 weeks. With the exception of BMI data (missing for 11 participants) there was minimal missing baseline data. During the study, 74 participants achieved complete healing, with 10 participants remaining unhealed at study end, 4 lost to follow-up, 9 withdrawn from clinic and 3 withdrawn from the study. Biological sampling was feasible with a mean of 5 samples collected per participant. Dressing practices were heterogeneous with 45 dressing types used. The median time taken from Biobank’s collection of the last dressing sample of the day, transport and processing before storage in the laboratory for all clinic sessions was 85 minutes.’
3. The definition of retention could still be clarified. Seventy-four participants reached the expected endpoint of complete healing, while the remaining participants had several different reasons for study exit. These should continue to be clearly distinguished from true loss to follow-up, rather than being grouped conceptually as attrition. The authors have improved this substantially, but consistency throughout the manuscript should be checked.	The definition of retention has now been clarified further. The feasibility question regarding retention has now been amended for further clarity: ‘How many people recruited to the study withdrew or were lost to follow-up?’<br>Throughout the manuscript, the definitions of reasons for study exit have been made clearer.<br>-Lost to follow-up meant that the participant did not return to clinic when they missed 4 clinic visits (8 weeks).<br>-Withdrawal from clinic was due to changes in ulcer management requirements such as discharge from

	clinical care, transferral to treatment rooms as compression therapy no longer required or receiving at home treatment.<br>-Withdrawal from the study was due to change in ulcer aetiology.
4. The absence of prespecified progression criteria remains an important limitation. Since no predefined recruitment, retention, data-completeness or sampling thresholds determined whether progression to a larger study would be considered feasible, the conclusions regarding feasibility are necessarily partly interpretative. This should remain clearly acknowledged when discussing progression to a definitive study.	Thank you for this helpful comment. We agree that the absence of prespecified progression criteria is an important limitation and that this should remain explicit in the manuscript. Our intention was to assess feasibility in an early-stage, exploratory sense, given the limited robust prospective evidence in this field, rather than to determine progression to a specific predefined definitive study. We have revised the manuscript to clarify that recruitment, retention, data completeness, and acceptability were assessed empirically to inform the design and planning of future research, rather than against formal go/no-go thresholds. We have also strengthened the limitations text to acknowledge that conclusions regarding feasibility are therefore partly interpretative and dependent on the aims, scale, and design of any subsequent study.
5. The claim regarding lack of disruption to routine clinical care should remain appropriately cautious. This was based on researcher observation and informal staff feedback rather than a formally measured outcome. The revised manuscript acknowledges this, and I recommend ensuring that wording such as "successful" does not inadvertently imply formally demonstrated acceptability or operational efficiency.	We have now removed any wording such as "successful" in relation to lack of disruption to routine clinical care and revised the text to ensure that the findings are presented more cautiously, reflecting that this observation was based on researcher experience and informal staff feedback rather than formal measurement.
6. The generalisability of the findings should be emphasised. The study was conducted in only three community leg-ulcer clinics in Greater Manchester and benefited from established Biobank infrastructure. The feasibility demonstrated here may therefore not translate directly to centres without similar research infrastructure, staffing or wound-care pathways.	Thank you for your comment. The generalisability of the findings has now been further emphasised in the Discussion section as highlighted in the manuscript: 'Participants were recruited from three community leg ulcer clinics within a single NHS Trust and therefore may not fully represent patients managed in other geographical areas, service models or healthcare systems. In addition, these clinics were associated with established Biobank infrastructure; consequently, the feasibility observed in this study may not be directly transferable to centres without comparable

	research infrastructure, staffing, or wound care pathways.’
7. A final language and grammatical review is recommended. There are still several awkward sentences in the revised text, particularly in the Abstract (for example, “It was reassuring that were able to collect…”). These can be corrected during final editing without altering the scientific content.	Language and grammar have now been reviewed, and some phrasing has been refined.
