# Supplementary material for: Feasibility of a prospective cohort study to support future investigation of prognostic factors for venous leg ulcer healing
Source: BMJ Open. 2026 Oct 12;16(10):e122867. doi: 10.1136/bmjopen-2026-122867 (PMC13662055; doi:10.1136/bmjopen-2026-122867)
Supplement: Supplementary file 2 [file bmjopen-16-10-s001.docx]

**Supplementary Figure S1. Dressing sampling of participants over time in each of three community leg ulcer clinics.** The bar chart represents the number of participants who had dressing samples collected each week during the course of the study. Study follow-up continued up to 62 weeks. The different colours show the number sampled from each of the three clinics.
