# Supplementary material for: Feasibility of a prospective cohort study to support future investigation of prognostic factors for venous leg ulcer healing
Source: BMJ Open. 2026 Oct 12;16(10):e122867. doi: 10.1136/bmjopen-2026-122867 (PMC13662055; doi:10.1136/bmjopen-2026-122867)
Supplement: Supplementary file 3 [file bmjopen-16-10-s002.docx]

**A longitudinal prognostic biomarker feasibility study for venous leg ulcer healing**

**1A. Clinical Protocol**

**Division of Musculoskeletal and Dermatological Sciences**

**School of Biological Sciences Faculty of Biology, Medicine and Health**

**University of Manchester**

**Researcher Key Contact Details**

**Principal Investigator**

Professor Jo Dumville – Jo.dumville@manchester.ac.uk

**Principal Investigator of the Complex Wounds @ Manchester Biobank**

Mr Adam Reid – Adam.reid@manchester.ac.uk

**Co-Investigator of the Complex Wounds @ Manchester Biobank**

Mr Jason Wong – Jason.k.wong@manchester.ac.uk

**Collaborator**

Professor Sarah Herrick – Sarah.herrick@manchester.ac.uk

**Complex Wounds @ Manchester Biobank Clinical Research Practitioners**

Faith Phiri – Faith.phiri@mft.nhs.uk

Rebecca Cleworth – Rebecca.cleworth@mft.nhs.uk

**Complex Wounds @ Manchester Biobank Clinical Trials Manager**

Lawrie Rogerson – Lawrie.rogerson@mft.nhs.uk

**University Research Nurse**

Dr Sara Ud-Din – Sara.ud-din@manchester.ac.uk

**Research Associate**

Joe Harvey – Joe.harvey@manchester.ac.uk

**Project Manager**

Gergana Ivanova – Gergana.ivanova@manchester.ac.uk

**History of Amendments**

| **Amendment & Date** | **Brief description of Amendments** | **Brief justification of amendment** |
| --- | --- | --- |
| Version: 1, Date: 09.12.2022 | Original Version | Not applicable |
| Version: 2, Date: 29.01.2024 | 1. Follow-up has been changed from 12 months to 3 years. 2. Removal of inclusion criteria stating that participants will be recruited from MFT clinics. 3. Removed mention of collecting from two clinics. 4. Wound measurement changed from grid to ruler measures. | 1. Longer follow-up period required as these wounds can take longer to monitor. 2. The plan is to expand recruitment outside of Greater Manchester and from other community clinics in different Trusts. 3. Multiple clinics will be accessed with approval. 4. The clinic nurses measure the wounds using a ruler method. |
| Version: 3, Date: 21.02.2024 | 1. Addition to inclusion criteria - To recruit patients who have the largest venous leg ulcers in the first instance and smaller wounds thereafter. 2. Addition to the exclusion criteria - Any participant who has already taken part in this current study and then healed but subsequently have returned and have a new ulcer or their ulcer has recurred. 3. Addition to follow-up data collection - Data will be collected retrospectively via the Manchester University NHS Foundation Trust EMIS and HIVE systems. 4. Addition of Researcher Key Contact Details section. | 1. Difficulties in following up small ulcers as they heal quickly. 2. We will not be monitoring recurrence of venous leg ulcers. 3. EMIS and HIVE will be accessed retrospectively to collect any missed or additional healing data. 4. To have a log of key research contacts and collaborators for this study. |
| Version 4, Date: 13.02.2025 | 1. Study end date added | 1. Adequate sample size has been reached |

**Synopsis**

Wound healing times can be highly variable for a variety of reasons, which can make it difficult to predict healing times for individual wounds. Therefore, the identification of prognostic biomarkers for healing would be valuable for patients and wound care practitioners. Wound fluid is an ideal sample type for prognostic biomarker investigations and a variety of methods have been used for its collection. We have developed and undertaken initial validation of collection method, which utilises extraction of wound fluid from dressings. We need to further validate our sampling and assay approach before scaling up into a larger prognosis study. We also need to test the feasibility of larger scale sample collection processes in the clinical setting. There are several practical feasibility issues that need to be systematically explored. Therefore, we will run a small pilot cohort study in the community setting to explore several important feasibility questions to inform the development of a larger study. Thus, the aim here is to undertake a pilot study that will establish the feasibility of conducting a future larger cohort study in venous leg ulcers to identify potential prognostic biomarkers.

1. **Background**

Wounds are a break in skin epithelial continuity, which exposes internal tissues to the external environment.^1^ Most wounds heal via an orderly set of overlapping phases. However, healing can be disrupted in a variety of ways leading to extended healing times. We refer to open wounds that heal by secondary intention as complex wounds. These wounds include leg ulcers (which are caused by damaged or disease leg veins and/or arteries); pressure ulcers (caused by unrelieved pressure or friction on part of the body) or foot ulcers (caused by vascular and neurological complications).^2,3^ These wounds display disruption of normal healing processes, resulting in a state of pathological inflammation.^4,5^

Venous leg ulcers are the most common type of leg ulcer, resulting from underlying chronic venous insufficiency.^6^ These ulcers negatively impact on those affected and are costly for health care systems to manage, largely because of frequent nursing care required, exacerbated by extended healing times.^7^ As the prevalence of venous leg ulcers increase with age^8,9^ these wounds are likely to become more common given the ageing trajectory of the UK population. Better understanding the healing trajectory of a wound is important to patients and health professionals alike, allowing more informed conversations and treatment planning.^7,10^

Potential prognostic indicators of wound healing are varied and includes:^11^

- Whole-person level measures such as age, body mass index and presence of co-morbidities
- Wound level measures such as wound area and duration
- And cellular level measures such as different biochemical and microbiological factors

Prognostic factors at all levels need to be measured simultaneously to both aid prognosis of wound healing and identify independent predictors of healing that may provide targets for treatment development.^12,13^ One potential biochemical prognostic biomarker is protein expression in the wound, which can be measured with various proteomic techniques often using wound fluid as the analytical sample.^14,15^

Wound fluid is a heterogeneous complex mixture which represents the wound microenvironment.^16^ This fluid is an ideal sample type for prognostic biomarker investigations as it can be sampled quickly and non-invasively.^17^ We have developed and validated a method for the collection of wound fluid from patient dressings, which is compatible with various proteomic assay methods. This combined sampling and assay method allows us to identify and analyse the abundances of specific proteins of interest in complex wound fluid. However, the efficacy of this method in measuring relevant changes in protein biomarker abundance over the course of wound healing is yet to be confirmed.

We need to validate our sampling and assay approach before scaling up into a larger prognosis study. We also need to test feasibility of larger scale sample collection processes in the clinical setting. There are a number of practical feasibility issues that need to be systematically explored. The previous research data was collected at an outpatient clinic in a hospital setting but this setting is not suitable for larger scale work because people only attend the out-patient clinic sporadically, as most of their care is delivered in the community. Therefore, we will run a small pilot cohort study in a community setting to explore a number of important feasibility questions to inform the development of a larger study.

2. **Research objectives**

To undertake a pilot study that will establish the feasibility of conducting a future larger cohort study in venous leg ulcers to identify potential prognostic biomarkers.

Feasibility questions that the study will address are:

a) Will patients agree to consent to a study of this type?
b) How many eligible patients can be recruited in a 12-month period from community clinic settings?
c) How long does it take to recruit a patient and collect the required baseline data?
d) What is the rate of participant attrition and reasons for attrition?
e) What co-morbidities do participants have?
f) How often are we able to collect dressing samples from participants? How frequently are participants seen by practitioners?
g) What type of dressings are commonly used in the community for these leg ulcers?
h) Do participant’s dressing types vary over time?
i) What is the incidence of wound infection in participants?
j) Are we able to collect, store and transfer dressings for protein extraction in a timely and secure way?
k) Are we able to assess clinical outcome data at baseline and repeatedly over time?
l) Can we effectively assess/record time to complete healing?
m) What is the per sample resource use for collection, storage and processing?
n) What translation needs will be required for this study? As part of the pilot, we will assess how many people for whom English is not the first language and /or who are unable to read the documentation and give informed consent. Where patients could not be recruited, we will investigate what translation needs will be required for the full study.

The outcomes of this pilot study will be vital for the planning and conduct of a larger clinical study to investigate complex wound prognostic biomarkers.

**3. Study design**

This study will follow the course of a prospective, feasibility study with data collection continuing for a maximum of 12 months and a minimum of 4 months. Participant medical history and demographic data will be collected at baseline along with collection of wound specific measurements. Wound area measurements will be recorded at baseline and then once a month thereafter until study completion. A wounds dressing will be collected at baseline and then every week until study completion. Key clinical events including ulcer healing (healing is defined as complete epithelial cover in the absence of a scab (eschar) with no dressing required) will be measured during the study alongside various feasibility factors. All Participants will be followed until they exit the study, or the study concludes.

4. **Sample size and eligibility**

We will aim to gather data from a minimum of 100 participants with the possibility to expand depending on participant availability, logistical factors, and other potential issues, which are being investigated as part of this study. We aim to gather a suitable amount of pilot data to inform sample size calculations and allow exploration of feasibility issues. Patients who meet the eligibility criteria below will be recruited from community clinics.

4.1. **Inclusion criteria:**
- Participants must have at least one venous leg ulcer*. The ulcer followed up will be known as the **reference ulcer** – this ulcer will be chosen as the largest ulcer by area in order to see signs of wound area change/ reduction. The participants with the largest ulcers will be recuited first and those with smaller ulcers thereafter.

**A venous leg ulcer is defined as any break in the skin which had either (1) been present for > 6 weeks or (2) occurred in a person with a history of venous leg ulceration. Ulcers were considered purely venous if, clinically, no other aetiology was suspected. The ulcer is required to be venous in appearance (i.e. moist, shallow, of an irregular shape) and was to lie wholly or partially within the gaiter region of the leg.*

- People who, in the opinion of the person who is recruiting the potential participant, can understand the requirements of the study and who can provide written and verbal informed consent.
- ≥ 18 years of age
  4.2. **Exclusion criteria:**
- People who are unwilling or unable to give informed consent.
- Previous or current enrolment in any study to evaluate a wound focussed treatment option 30 days prior to entry in the study (the details of the other study will be discussed, and a decision made if that patient can be recruited).
- Any participant who has already taken part in this current study and then healed but subsequently have returned and have a new ulcer or their ulcer has recurred.
- People who are currently pregnant
- Non-English-speaking people
- People less than 18 years of age

5. **Recruitment**

Potential participants will be recruited from community clinics with an active leg ulcer service. The clinical nurse leads at the clinics will initially liaise with a Complex Wounds Biobank research nurse as to any potentially eligible patients. The research nurse will then attend the clinic, complete the pre-screening form to check eligibility and if eligible, they will then provide the potential participant with full information regarding the study, and give them a written information sheet for them to take their time to read (a minimum of 24 hours). If eligible, the following week at their next appointment, the potential participant will be asked again by the research nurse if they wish to take part in the study and if they are happy to participate, full written informed consent will be taken. Maintenance of confidentiality and compliance with the UK Data Protection Acts will be emphasised to all potential study participants. Participation in the study will be voluntary. Consent will only be taken once at the study start. All data will be treated with the strictest confidence. All research staff obtaining informed consent and collecting clinical data will undergo good clinical practice training. Pseudo-anonymisation of participants will take place at the point of data collection, all participants’ information and samples will have unique identifiers assigned to them. All participants will receive a unique sample identification number by the Biobank team. Within the Manchester University NHS Foundation Trust Biobank facility itself, the identification number for a sample is generated sequentially by the StarLiMS software and is not derived from any identifiable information of that participant. The identifier will be used as a unique ID for any samples released from the Biobank.

6. **Clinical procedure:**

- All collected data will be recorded by the research nurse using the REDCap data collection tool

**6.1	Day 0 data collection:**

-Following consent, our trained research nurse will collect the following data:

1. participants demographic data including age/date of birth, gender, ethnicity, height (cm), weight (kg)
2. Wound duration (months) at study entry (day 0)
3. Wound area measurement (cm^2^) - using a simple ruler method.
4. Infection status
5. Dressing name and brand
6. Dressing duration in-situ (hours)
7. A basic medical history will also be taken at this point; this will include previous and/or current medical conditions, mobility and smoking status.
8. Leg assessment data

- A diagram of the leg and the position of the reference leg ulcer will be recorded as per below.

- After the clinic nurse has removed the participant’s dressings, the whole dressings will then be collected as part of a standard dressing change and placed on ice by the research nurse or practitioner. This will be carried out adhering to Community NHS infection prevention policies and procedures. A new dressing will then be placed on the wounds by their regular NHS clinical nursing staff and standard care will resume.

- Dressings will then be transferred and processed as per “Transportation and Storage” in Section 7. The Experimental Protocol (1b) will then be followed.

**6.2. Follow up data collection:**

*Dressings*

Further dressing collections will be carried out by the research nurse or practitioner once every other week at follow-up clinical visits unless otherwise stated. The frequency of collection may change as the study progresses. The frequency of dressing changes will be recorded for both scheduled dressing collections and any additional dressing changes at the clinic. The name and brand of the wound dressing taken for sampling will be recorded at each collection.

*Clinical data*

-Wound area measurements will be taken once weekly by the clinic nurse staff.

-Any changes to clinical status for study relevant events:

- Reference ulcer infection (in the opinion of the treating nurse, with a record of signs and symptoms requested)
- A healed reference ulcer, where a healed ulcer is defined as complete epithelial cover in the absence of a scab (eschar) with no dressing required (see section 3)
- Death
- Admission to hospital

-Data will be collected directly or from participant notes by the research nurse or practitioner at follow up visits as required.

-Data will also be collected retrospectively via the Manchester University NHS Foundation Trust EMIS and HIVE systems to collect any missed or additional healing data.

**6.3 Duration of follow-up**

Participants will be followed for clinical data and/or sample collection from a minimum of 4 months until a maximum of 12 months or the conclusion of the study or until study exit. The final people were recruited for dressing sample collections on 19^th^ December 2024, therefore, all dressing sample collection will cease 4 months after this date on 19^th^ April 2025. The justification for this is that we have already collected from over 100 patients, so we are now stopping recruitment for samples. The 4 month time point was chosen based on predicted event rates and a median time to ulcer healing of 3-4 months (Westby et al, 2016 #20).

**6.3 Study exit**

Data will be collected on participants until they exit the study, for one of the following reasons:

- Participant withdraws from the study requesting cessation of all data collection
- Participant dies
- Participant is lost to follow-up
- Participants’ reference ulcer heals
- Participant is non-healing by 12 months
- End of study

**Summary Tables**

**Table 1: Patient Pre-screening questions: Reasons for study exclusion will be recorded**

- Does the potential participant have at least one venous leg ulcer?
- Is the potential participant over the age of 18 years?
- Is the potential participant able to understand the requirements of the study and provide written and verbal informed consent?
- Can the potential participant speak English and understand the requirements of the study?
- Has the potential participant had any previous or current enrolment in any study to evaluate a wound focussed treatment option 30 days prior to entry in this study?
- Is the potential participant currently pregnant?

| **Table 2: Participant data collected at baseline (Day 0)** | |
| --- | --- |
| **Participant demographics** | - Date of birth, gender, ethnicity, height, weight - Location of clinic |
| **Leg ulcer assessment and history** | - Number of leg ulcers - Location of reference leg ulcers - Reference leg ulcer duration at study entry - Wound area measurement of reference leg ulcer - Participants’ level of mobility |
| **Co-morbidities** | - Smoking status - Cardiovascular disease - Peripheral arterial disease - Diabetes - Stroke - Cancer - Other (please list) |
| **Infection status** | - Any presence/absence of wound infection of the reference ulcer at baseline and how infection was assessed |
| **Dressings** | - Name of dressing used - Duration of dressing *in situ* - Number of dressing changes per week |
| **Current compression Treatment** | - Four layer compression bandage - Reduced compression bandaging - Two layer bandage - Short stretch bandage - Single layer compression hosiery - Two layer compression hosiery - Compression wraps - Other compression bandaging - Other compression hosiery - Other compression wrap - Not currently receiving compression |
| **Surgical treatment (and dates)** | - Endovenous ablation - Foam sclerotherapy - High tie and stripping - Avulsions - Other form of surgery |

| **Table 3: Participant data collected at follow-up visits** | |
| --- | --- |
| **Follow-up visits** | - Assume no changes to status. If any changes to clinical data, such as wound infection record in REDCap. - Wound area measurement – each visit - Dressing name and duration of use – each visit |

7. **Transportation and storage:**

Dressing samples will be transported by car by the University research nurse, on ice, in a biohazard vessel bag to the laboratory research assistant at the research facilities in the Stopford Building at the University of Manchester on the day of collection. Transportation and storage will adhere to Human Tissue Act guidelines and University of Manchester policies and guidance. The research assistant will cut the dressings into 2cm² sections and store securely in the laboratory -80°C freezer until processing and analysis. The samples will be anonymised (coded) by the Biobank Clinical Trials Coordinator and/or Research Practitioner; therefore, no other personnel outside of the Biobank team will be able to trace the identity of the sample (except research monitors to ensure the appropriate procedures are being followed). As per University of Manchester guidelines, data will be retained for 10 years.

8. **Outcomes and analysis**

The primary aim of this pilot study is to assess the feasibility and logistics of conducting a larger study in the future. The primary outcome of the main study is likely to be time to healing, so we will collect data on time to complete healing. This pilot study will aim to provide us with much important information that will dictate the design of the full study. We will collect data with the aim of addressing the above feasibility questions set out in section 2 and will report findings at the end of the pilot study.

*Clinical outcome data*

The area of the reference ulcer will be measured weekly. We will record when the reference ulcer is healed. We will also record when the reference ulcer is diagnosed as infected by the treating nurse. Healing is defined as complete epithelial cover in the absence of a scab (eschar) with no dressing required. We will not conduct blinded outcome assessment, which is less of an issue in this non-interventional study.

| **Table 4: Data collection processes for outcome data to be collected during the study*. *In all cases missing data will be reported and not imputed and will not be included in denominator values in relevant calculations.*** | | |
| --- | --- | --- |
| **Research question** | **Relevant data collection and measurements** | **Analyses*** |
| a) Will patients agree to consent to a study of this type?   And     b) How many eligible patients can be recruited from these two community clinic settings? | Data collected at pre-study screening phase   - Total number of patients attending each clinic alongside total number who agree to take part - Number of eligible patients measured, from those who agree to take part, in pre-study screening against inclusion criteria during recruitment period - Reasons for ineligibility recorded | Descriptive statistics for the number of people screened, eligible and recruited presented as proportions. Conversion from eligible to recruited presented.  Reasons for ineligibility (by each criterion) presented. |
| c) How long does it take to recruit a patient and collect the required baseline data? | Data collected during screening, recruitment, and baseline data collection process   - Cumulative time taken for the screening, recruitment and baseline data collection | Data presented as mean time taken for each stage with standard deviation. Median data with inter-quartile range also presented |
| d) What is the rate of attrition and reasons for attrition? | - The length of time each participant took part in the study will be calculated as the difference between date of consent and date of study exit - Reasons for withdrawal and clinical events documented and quantified by category | Number and proportion of participants that leave the study before healing (or study completion) and reasons for this. |
| e) What are the participants’ co-morbidities in this eligible and consenting population? | Documentation of previous and/or current co-morbidities based on list of key co-morbidities   - Co-morbidities collected from participant notes - Quantification of individual co-morbidities | Number of participants with relevant co-morbidities by category with proportions given for each. Presented in tabular format with amount of missing data recorded. |
| f) How often are we able to collect dressing samples from people with venous leg ulcers in the community setting? How frequently are community patients seen by practitioners? | Frequency of dressing changes   - This will be calculated from the number of times the dressings are collected at the clinics by the research nurse and any additional dressing changes at the clinic outside scheduled collections - Some data may be collected retrospectively; the research nurse may consult participants’ notes or records to determine the number of dressing changes over a specified period. - Missed collections for participants recorded as necessary | Descriptive statistics for frequency of dressing changes with mean averages and standard deviations given |
| g) What type of dressings are commonly used in the community for venous leg ulcers?   AND   h) Do dressings used by participants change over time? | - Name and material of dressing will be recorded on collection of the dressing - Changes to dressing type will be noted - This information will be recorded at each visit | Number of times each dressing type used and proportions for each. Missing data also recorded.  Changes to dressing types used (if any) over time visualised in graphical format. |
| i) What is the incidence of wound infection? | - Infection status will be assessed at baseline and at each clinic visit by the clinic nurses who will then inform the research nurse of the participants’ infection status. | Descriptive statistics for number of people infected and not infected at baseline and at monthly intervals throughout the study.  Changes over time visualised graphically. |
| j) Are we able to collect, store and transfer dressings for protein extraction in a timely and secure way? | - Record time taken for collection and transport before storage in -80°C freezer - Record any issues with collection and transport which cause delays | Mean average calculated for collection and storage times and assessed against relative literature. |
| k) Are we able to assess demographic and clinical outcome data at baseline and repeatedly over time? | - Collection of data at baseline and at follow up sessions - Record of missing data | Proportions for data collected and for data missed/not collected. |
| l) Can we effectively assess/record time to complete healing? | Collection of healing data from study participants   - The date the community nurse considered the reference leg ulcer to have healed will be recorded - Time to healing of participants’ reference leg ulcer will utilise data of study entry, date of healing or date of study exit, whichever comes first. | We will record the number of people with a date of healing and present this as a proportion of participants alongside the amount of missing data.  Whist the main aim of this feasibility study is not to calculate time to healing per se, we will present these data for descriptive information alongside measures of uncertainty around the estimates.  Time to healing will be calculated using standard time to event analyses accounting for censoring. Data will be presented as median time to healing if possible (that is if 50% or more of participants heal during the study). We will also present data as a hazard rate, via use of a Kaplan-Meier curve, which presents the instantaneous rate of the event at each point in time. |
| m) What is the per sample resource use for collection, storage and processing | - This will be calculated in an excel spreadsheet based on Biobank costs, staff costs, transport, storage, laboratory consumables. - Cost of all materials will be quantified and tabulated | Total costs will be divided by either number of participant or number of samples to give average cost per participant and per sample collected. |
| n) What translation needs are required for this study and what proportion of future participants is likely to require translation of documentation into which language? | Number of participants who require translation of documents and other information   - Number of potential participants whose first language is not English for whom use of written English is prohibits them from taking part in the study. - Record made of primary languages spoken and read where possible | Proportion of potential participants who did not speak English and quantification of primary languages spoken by participants |

9. **Data transfer**

The data will be anonymised (coded) by the Biobank Clinical Trials Coordinator and/or Research Practitioner; therefore, no other personnel outside of the Biobank team will be able to trace the identity of the sample (except research monitors to ensure the appropriate procedures are being followed). As per University of Manchester guidelines, data will be retained for 10 years. All data will be collected in the REDCap data collection tool electronically. REDCap is a secure web application for building and managing online surveys and databases. The use of REDCap is through the license agreement with Manchester University NHS Foundation Trust. When samples are released to the end user only non-identifiable data will be sent. Justification must be provided for all data requested. As with access to samples, access to data will involve review of the application from researchers to the Biobank Ethics Advisory Group. A separate form is required documenting the clinical data required, method of data transfer and justification for such data being released. All personal identifiers are removed and only the anonymised participant (CWB number) & sample identification number (as recorded on the Biobank database) is used. This code cannot be used by the recipient to identify the donor. In addition, the MTA signed by all recipient parties’ states that they must not under any circumstances attempt to identify the donor(s). The Biobank coordinator or Research Nurse/Practitioner will then extract the requested and approved anonymised data from REDCap and provide it to the researchers using the method documented on the application (electronic transfer to academic/professional email address).

**References**

1) Fonder MA, Lazarus GS, Cowan DA, Aronson-Cook B, Kohli AR, Mamelak AJ. Treating the chronic wound: A practical approach to the care of nonhealing wounds and wound care dressings. J Am Acad Dermatol. 2008 Feb;58(2):185-206. doi: 10.1016/j.jaad.2007.08.048. PMID: 18222318.
2) Guo R, Xu S, Ma L, Huang A, Gao C. Enhanced angiogenesis of gene-activated dermal equivalent for treatment of full thickness incisional wounds in a porcine model. Biomaterials. 2010 Oct;31(28):7308-20. doi: 10.1016/j.biomaterials.2010.06.013. Epub 2010 Jul 2. PMID: 20598366.
3) Ferreira MC, Tuma P Jr, Carvalho VF, Kamamoto F. Complex wounds. Clinics (Sao Paulo). 2006 Dec;61(6):571-8. doi: 10.1590/s1807-59322006000600014. PMID: 17187095.
4) Agren MS, Eaglstein WH, Ferguson MW, Harding KG, Moore K, Saarialho-Kere UK, Schultz GS. Causes and effects of the chronic inflammation in venous leg ulcers. Acta Derm Venereol Suppl (Stockh). 2000;210:3-17. PMID: 10884942.
5) Maschalidi S, Mehrotra P, Keçeli BN, De Cleene HKL, Lecomte K, Van der Cruyssen R, Janssen P, Pinney J, van Loo G, Elewaut D, Massie A, Hoste E, Ravichandran KS. Targeting SLC7A11 improves efferocytosis by dendritic cells and wound healing in diabetes. Nature. 2022 Jun;606(7915):776-784. doi: 10.1038/s41586-022-04754-6. Epub 2022 May 25. Erratum in: Nature. 2022 Aug;608(7923):E29. doi: 10.1038/s41586-022-05101-5. PMID: 35614212.
6) Vasudevan B. Venous leg ulcers: Pathophysiology and Classification. Indian Dermatol Online J 2014;5(3):366-70. doi: 10.4103/2229-5178.137819
7) Urwin S, Dumville JC, Sutton M, Cullum N. Health service costs of treating venous leg ulcers in the UK: evidence from a cross-sectional survey based in the north west of England. BMJ Open 2022;12(1):e056790. doi: 10.1136/bmjopen-2021-056790 [published Online First: 20220106]
8) Hall J, Buckley HL, Lamb KA, et al. Point prevalence of complex wounds in a defined United Kingdom population. Wound Repair Regen 2014;22(6):694-700. doi: 10.1111/wrr.12230 [published Online First: 20150108]
9) Gray TA, Rhodes S, Atkinson RA, et al. Opportunities for better value wound care: a multiservice, cross-sectional survey of complex wounds and their care in a UK community population. BMJ Open 2018;8(3):e019440. doi: 10.1136/bmjopen-2017-019440 [published Online First: 20180322]
10) Hemingway H, Croft P, Perel P, et al. Prognosis research strategy (PROGRESS) 1: a framework for researching clinical outcomes. Bmj 2013;346:e5595. doi: 10.1136/bmj.e5595 [published Online First: 20130205]
11) Westby MJ, Dumville JC, Stubbs N, Norman G, Wong JK, Cullum N, Riley RD. Protease activity as a prognostic factor for wound healing in venous leg ulcers. Cochrane Database Syst Rev. 2018 Sep 1;9(9):CD012841. doi: 10.1002/14651858.CD012841.pub2. PMID: 30171767; PMCID: PMC6513613.
12) Warriner RA, Snyder RJ, Cardinal MH. Differentiating diabetic foot ulcers that are unlikely to heal by 12 weeks following achieving 50% percent area reduction at 4 weeks. Int Wound J. 2011 Dec;8(6):632-7. doi: 10.1111/j.1742-481X.2011.00860.x. Epub 2011 Sep 23. PMID: 21951763; PMCID: PMC7950518.
13) Ladwig GP, Robson MC, Liu R, Kuhn MA, Muir DF, Schultz GS. Ratios of activated matrix metalloproteinase-9 to tissue inhibitor of matrix metalloproteinase-1 in wound fluids are inversely correlated with healing of pressure ulcers. Wound Repair Regen. 2002 Jan-Feb;10(1):26-37. doi: 10.1046/j.1524-475x.2002.10903.x. PMID: 11983004.
14) Broszczak D SD, Compay ALL, et al. Biochemical profiling of proteins and metabolites in wound exudate from chronic wound environments. Wound Practice and Research 2012;20(2):62-72.
15) Edsberg LE, Wyffels JT, Brogan MS, Fries KM. Analysis of the proteomic profile of chronic pressure ulcers. Wound Repair Regen 2012;20(3):378-401. doi: 10.1111/j.1524-475X.2012.00791.x
16) Yager DR, Kulina RA, Gilman LA. Wound fluids: a window into the wound environment? Int J Low Extrem Wounds. 2007 Dec;6(4):262-72. doi: 10.1177/1534734607307035. PMID: 18048872.
17) Saleh K, Strömdahl AC, Riesbeck K, Schmidtchen A. Inflammation Biomarkers and Correlation to Wound Status After Full-Thickness Skin Grafting. Front Med (Lausanne) 2019;6:159. doi: 10.3389/fmed.2019.00159 [published Online First: 20190712]
