# Supplementary material for: Feasibility of a prospective cohort study to support future investigation of prognostic factors for venous leg ulcer healing
Source: BMJ Open. 2026 Oct 12;16(10):e122867. doi: 10.1136/bmjopen-2026-122867 (PMC13662055; doi:10.1136/bmjopen-2026-122867)
Supplement: Supplementary file 4 [file bmjopen-16-10-s003.docx]

**Supplementary Material 2**

*Data Transfer*

The data was anonymised by the Biobank Clinical Trials Coordinator, therefore, no other personnel outside of the Biobank team was able to trace the identity of the sample. All data was collected in the REDCap data collection tool electronically. REDCap is a secure web application for building and managing online surveys and databases. The use of REDCap was through the license agreement with Manchester University NHS Foundation Trust. When samples were released to the end user (The University of Manchester), only non-identifiable data was sent. As with access to samples, access to data involved a review of the application from the University of Manchester research team to the Biobank Ethics Advisory Group. A separate form was required documenting the clinical data required, method of data transfer and justification for such data being released. The Biobank coordinator or Research Practitioner extracted the requested and approved anonymised data from REDCap and provided it to the researchers by electronic transfer.
