# Supplementary material for: Feasibility of a prospective cohort study to support future investigation of prognostic factors for venous leg ulcer healing
Source: BMJ Open. 2026 Oct 12;16(10):e122867. doi: 10.1136/bmjopen-2026-122867 (PMC13662055; doi:10.1136/bmjopen-2026-122867)
Supplement: Supplementary file 5 [file bmjopen-16-10-s004.docx]

**Supplementary Table S1 | Participant data collected at baseline and follow-up visits**

| **Participant data collected at baseline (Day 0)** | |
| --- | --- |
| **Participant demographics** | - Date of birth, sex, ethnicity, height, weight - Location of clinic |
| **Leg ulcer assessment and history** | - Number of leg ulcers - Location of reference leg ulcers - Reference leg ulcer duration at study entry - Wound area measurement of reference leg ulcer - Participants’ level of mobility |
| **Co-morbidities** | - Smoking status - Cardiovascular disease - Peripheral arterial disease - Diabetes - Stroke - Cancer - Other (please list) |
| **Infection status** | - Any presence/absence of wound infection of the reference ulcer at baseline and how infection was assessed |
| **Dressings** | - Name of dressing used - Duration of dressing *in situ* - Number of dressing changes per week |
| **Current compression Treatment** | - Four-layer compression bandage - Reduced compression bandaging - Two-layer bandage - Short stretch bandage - Single layer compression hosiery - Two-layer compression hosiery - Compression wraps - Other compression bandaging - Other compression hosiery - Other compression wrap - Not currently receiving compression |
| **Surgical treatment (and dates)** | - Endovenous ablation - Foam sclerotherapy - High tie and stripping - Avulsions - Other form of surgery |

| **Participant data collected at follow-up visits** | |
| --- | --- |
| **Follow-up visits** | - Assume no changes to status. If any changes to clinical data, such as wound infection record in REDCap. - Wound area measurement – each visit - Dressing name and duration of use – each visit |

**Supplementary Table S2 | Screening, recruitment and baseline data collection times for a sample of 20 participants**

| **Screening time (minutes)** | **n=20** |
| --- | --- |
| Mean (SD) | 3.8 (0.7) |
| Median (IQR) | 4 (1.0) |
| Missing data | 0 |
| **Recruitment time (minutes)** | **n=20** |
| Mean (SD) | 9.8 (1.8) |
| Median (IQR) | 9.5 (3.3) |
| Missing data | 0 |
| **Baseline data collection time (minutes)** | **n=20** |
| Mean (SD) | 10.3 (2.3) |
| Median (IQR) | 10 (3.3) |
| Missing data | 0 |
| **Cumulative time taken (minutes)** | **n=20** |
| Mean (SD) | 23.8 (3.1) |
| Median (IQR) | 24.5 (4.0) |
| Missing data | 0 |

**Supplementary Table S3 | Incidence of wound infection during the course of the study period.**

| **Infection status at baseline** | **n=100** |
| --- | --- |
| Infected (%) | 6 (6) |
| Not infected (%) | 94 (94) |
| Missing data (%) | 0 (0) |
| **Type of infection at baseline** | **n=6** |
| *Pseudomonas aeruginosa* (%) | 4 (66.6) |
| *Streptococcus dysgalactiae* (%) | 1 (16.7) |
| Missing data (%) | 1 (16.7) |
| **Incidence of infection during study period** | **n=100** |
| Incidence of infection (%) | 6 (6) |
| No new incidence of infection (%) | 94 (94) |
| Missing data (%) | 0 (0) |
| **Type of infection during the study period** | **n=6** |
| *Pseudomonas aeruginosa* (%) | 3 (50) |
| Missing data (%) | 3 (50) |

**Supplementary Table S4 | Venous leg ulcer dressings information used during the study.**

|  | **Clinic 1** |  | **Clinic 2** | **Clinic 3** |  |  | **All clinics** |
| --- | --- | --- | --- | --- | --- | --- | --- |
| **Frequency of dressing changes per week** | **n=216** |  | **n=41** | **n=246** |  |  | **n=503** |
| 1 (%) | 172 (79.6) |  | 40 (97.6) | 184 (74.8) |  |  | 396 (78.7) |
| 2 (%) | 38 (17.6) |  | 1 (2.4) | 62 (25.2) |  |  | 101 (20.1) |
| 3 (%) | 5 (2.3) |  | 0 (0.0) | 0 (0.0) |  |  | 5 (1.0) |
| Missing data (%) | 1 (0.5) |  | 0 (0.0) | 0 (0.0) |  |  | 1 (0.2) |
| **Dressings collected per participant** | **n=215** |  | **n=41** | **n=245** |  |  | **n=501** |
| Mean (SD) | 8.6 (9.1) |  | 4.1 (3.3) | 3.8 (3.2) |  |  | 5.1 (5.7) |
| Median (IQR) | 5 (7.0) |  | 3.5 (5.0) | 3 (3.0) |  |  | 3 (4.0) |
| **Dressing types^a^** | **n=346** |  | **n=64** | **n=348** |  |  | **n=758** |
| Kliniderm superabsorbant (%) | 106 (30.6) |  | 22 (34.4) | 60 (17.2) |  |  | 188 (24.8) |
| Aquacel Ag+ (%) | 44 (12.7) |  | 4 (6.3) | 17 (4.9) |  |  | 65 (8.6) |
| Aquacel Extra (%) | 37 (10.7) |  | 22 (34.4) | 12 (3.4) |  |  | 71 (9.4) |
| Atrauman Ag | 10 (2.9) |  | 0 (0.0) | 13 (3.7) |  |  | 23 (3.0) |
| Kliniderm foam (%) | 11 (3.2) |  | 4 (6.3) | 23 (6.6) |  |  | 38 (5.0) |
| Polymem (%) | 42 (12.1) |  | 2 (3.1) | 15 (4.3) |  |  | 59 (7.8) |
| Atrauman (%) | 14 (4.0) |  | 2 (3.1) | 69 (19.8) |  |  | 85 (11.2) |
| Urgostart plus (%) | 7 (2.0) |  | 0 (0.0) | 19 (5.5) |  |  | 26 (3.4) |
| Unknown (%) | 14 (4.0) |  | 2 (3.1) | 7 (2.0) |  |  | 23 (3.0) |
| Other (%)^b^ | 60 (17.3) |  | 6 (9.4) | 122 (32.2) |  |  | 178 (23.5) |
| None (%) | 1 (0.3) |  | 0 (0.0) | 1 (0.3) |  |  | 2 (0.3) |
| **Number of dressing types used** |  |  |  |  |  |  | **n=100** |
| 1 (%) |  |  |  |  |  |  | 29 (29) |
| 2 (%) |  |  |  |  |  |  | 17 (17) |
| 3 (%) |  |  |  |  |  |  | 19 (19) |
| 4 (%) |  |  |  |  |  |  | 12 (12) |
| 5 (%) |  |  |  |  |  |  | 10 (10) |
| >5 (%) |  |  |  |  |  |  | 13 (13) |

**Supplementary Table S5 | Dressing collection, transport and storage time (minutes)**

| **All clinics** |  |
| --- | --- |
| Total number of visits (%) | 118 (100.0) |
| Mean (SD) (minutes) | 111.4 (70.9) |
| Median (IQR) (minutes) | 85 (46.0) |
| Missing data (%) | 2 (1.7) |
| **Clinic 1** |  |
| Total number of visits (%) | 44 (37.3) |
| Mean (SD) (minutes) | 82.2 (18.2) |
| Median (IQR) (minutes) | 80.5 (19.8) |
| Missing data (%) | 0 |
| **Clinic 2** |  |
| Total number of visits (%) | 9 (7.6) |
| Mean (SD) (minutes) | 107.1 (77.5) |
| Median (IQR) (minutes) | 75 (55.0) |
| Missing data (%) | 0 |
| **Clinic 3** |  |
| Total number of visits (%) | 63 (53.4) |
| Mean (SD) (minutes) | 133.1 (85.6) |
| Median (IQR) (minutes) | 92 (110.0) |
| Missing data (%) | 2 (1.7) |
